# Differential Expression of Woodchuck Toll-Like Receptors 1–10 in Distinct Forms of Infection and Stages of Hepatitis in Experimental Hepatitis B Virus Infection

**DOI:** 10.3389/fmicb.2018.03007

**Published:** 2018-12-07

**Authors:** John Bradley Williams, Alena Hüppner, Patricia M. Mulrooney-Cousins, Tomasz I. Michalak

**Affiliations:** Molecular Virology and Hepatology Research Group, Division of BioMedical Sciences, Faculty of Medicine, Health Sciences Centre, Memorial University of Newfoundland, St. John’s, NL, Canada

**Keywords:** woodchuck model of hepatitis B, hepadnaviral infection, toll-like receptors, liver, hepatocytes, PBMC, lymphatic organs

## Abstract

Woodchucks infected with woodchuck hepatitis virus (WHV) represent a highly valuable model of human hepatitis B virus (HBV) infection, chronic hepatitis (CH), and virus induced-primary liver cancer. Toll-like receptors (TLRs) are important mediators of immune responses playing pivotal roles in the pathogenesis of viral diseases; however, their expression profiles in different forms of infection and stages of hepatitis, and in healthy animals remain essentially unknown. In this study, woodchuck TLRs 1–10 exon fragments were identified and TLR genes transcription quantified in livers, primary hepatocytes, peripheral blood mononuclear cells (PBMC), and in selected organs during experimental WHV infection. Among others, liver biopsies from acute hepatitis (AH) and CH showed significantly augmented expression of the majority of TLRs when compared to healthy and woodchucks prior to AH, with resolved AH or primary occult infection. In contrast to the liver tissue, significant upregulation of TLR3, TLR4, and TLR10, but downregulation of TLR7, characterized hepatocytes derived from livers of animals with resolved AH accompanied by secondary occult infection. Hepatocytes from CH showed significantly lower expression or a trend toward suppression of several TLRs when compared to hepatocytes from healthy animals and woodchucks with other forms of infection or hepatitis, suggesting that hepatocyte innate immune response is downregulated during CH. Contrastingly, upregulated transcription of some TLRs characterized PBMC throughout CH. Our study uncovered that TLR expression significantly varies between different forms of hepadnaviral infection and whether infection is accompanied or not by hepatitis. The results showed that the profiles of TLRs’ expression in circulating lymphomononuclear cells do not mirror accurately those of livers and hepatocytes from infected animals. These findings are of importance to the understanding of immune process operating at different sites targeted by virus in the course of hepadnaviral infection and evaluation of future therapies modifying antiviral innate responses in the woodchuck model.

## Introduction

An estimated 257 million people worldwide have chronic hepatitis (CH) caused by hepatitis B virus (HBV) and up to a million die annually as a consequence of infection with this virus (World Health Organization, 2018). In addition, up to two billion may have occult infection persisting in the absence of detectable HBV surface antigen (HBsAg) in serum and clinical manifestations of liver disease. Symptomatic HBV infection normally begins as acute hepatitis (AH) that spontaneously resolves in the majority of adults and, therefore, is denoted as self-limited AH (SLAH or SL). Importantly, SLAH is followed by occult HBV persistence, designated as secondary occult infection (SOI), which progresses in the absence of clinically detectable serum HBsAg, however, antibodies to HBV core antigen (anti-HBc), and low levels of circulating HBV DNA and virus replication in the liver and in peripheral blood mononuclear cells (PBMC) are identifiable (Michalak et al., 1994; Raimondo et al., 2008). In 5–10% of cases, AH advances to CH that is accompanied by serum HBsAg positivity, a high HBV DNA load (up to 10^10^–10^11^ genome copies or virus genome equivalents [vge]/mL) and by biochemical and histological indicators of protracted liver inflammation (Ganem and Prince, 2004; McMahon, 2010). CH can advance to liver cirrhosis and hepatocellular carcinoma (HCC), which is the major cause of death among patients with CH type B and has a dismal 5-year survival (Yang and Roberts, 2010; Ryerson et al., 2016). Despite prophylactic vaccines, the global reservoir of HBV is not substantially decreasing, mainly due to perinatal virus transmission, many unvaccinated children, unsafe intravenous drug usage, and migration.

Hepatitis B virus is the prototype of a class of the smallest, partially double-stranded DNA, non-cytopathic viruses named hepadnaviruses (Seeger and Mason, 2015). Their unique characteristic is reverse transcription of genomic DNA to RNA and RNA back to DNA. As consequence, these viruses are highly prone to mutations, but they also have features common with retroviruses regarding the reverse transcription step and that their DNA irreversibly integrates to the host’s genome (Nassal, 2008; Mulrooney-Cousins et al., 2014; Levrero and Zucman-Rossi, 2016). This virus family includes pathogens infecting human, woodchuck, ground squirrel, tree squirrel, bat (mammalian viruses), and duck, heron and snow geese (avian viruses). Among them, woodchuck hepatitis virus (WHV) displays the greatest compatibility to HBV considering genome sequence homology (∼65%), antigenic cross-reactivity, spectrum of organs targeted (i.e., liver and immune system), the patterns of cellular and humoral immune responses, the sequence of liver disease progression, and susceptibility to anti-viral therapy (Michalak, 1998; Tennant et al., 2004; Menne and Cote, 2007; Roggendorf et al., 2015). Eastern North American woodchucks (*Marmota monax*) infected with WHV develop AH that can advance to CH and HCC or progress from AH to SLAH/SOI and HCC or develop primary occult infection (POI) that can also advance to liver cancer (Korba et al., 1989; Michalak et al., 1999; Michalak, 2000; Mulrooney-Cousins et al., 2014; Mulrooney-Cousins and Michalak, 2015). Animals inoculated with very low doses of WHV (<10^3^ virions) develop a primary asymptomatic persistent infection, designated as POI (Michalak et al., 2004). This infection coincides with life-long replication of trace amounts of infectious WHV that occurs in the absence of detectable serum WHV surface antigen (WHsAg), antibodies to WHV core antigen (anti-WHc) and antibodies to WHsAg (anti-WHs). Nonetheless, low levels of virus genome are detectable in the circulation and infected organs at levels <100–200 vge/ml and <10^3^ vge/μg of total DNA, respectively, while WHV RNA in PBMC and livers occurs at estimated levels of <10^3^ vge copies per μg of total RNA (Michalak et al., 2004; Mulrooney-Cousins et al., 2014; Mulrooney-Cousins and Michalak, 2015). Interestingly, WHV-specific proliferative and cytotoxic T cell responses display similar characteristics in both POI and SOI (Gujar et al., 2008; Gujar and Michalak, 2009). In contrast to SOI, POI is not accompanied by virus-specific B cell reactivity and does not provide protection against reinfection with WHV (Mulrooney-Cousins and Michalak, 2015). A form of HBV infection similar to WHV POI has been reported in humans (Zerbini et al., 2008). Notably, HCC develops in similar frequency (∼20%) in woodchucks with either SOI or POI, clearly demonstrating pathogenic importance of these two distinct forms of occult hepadnaviral persistence (Michalak et al., 1999; Mulrooney-Cousins et al., 2014). Overall, WHV infection in woodchucks represents the closest pathogenic model of HBV infection, hepatitis B and HBV-associated HCC. It also is the most valuable, naturally occurring system for *in* *vivo* evaluations of novel anti-HBV and anti-HCC therapies, including those based on the approaches modifying hepatic innate immunity involving Toll-like receptors (TLRs).

Due to the non-cytopathic nature of hepadnaviruses, virus-triggered immune responses are primarily responsible for induction of hepatocyte injury and liver inflammation, as well as for resolution or persistence of hepatitis. TLRs are innate sensors belonging to the pattern recognition system identifying conserved motifs of microorganisms (Rock et al., 1998; Aderen and Ulevitz, 2000). In microbial infections, they sense non-self components and transmit signals that activate a range of cytokines downstream. These cytokines subsequently trigger cells of the innate immune system and activate effectors of the adaptive immune response, i.e., T and B cells (Mencin et al., 2009; Blasius and Beutier, 2010; Hua and Hou, 2013). Therefore, the interaction between virus and TLRs is considered critical to viral pathogenicity, particularly in infections with non-cytopathic viruses.

As indicated, HBV and WHV can initiate different forms of infection and the induced hepatitis may advance to chronic liver disease and HCC or resolve, although virus never completely clears (Michalak et al., 1994, 1999). In this context, identification of the TLR expression profiles in virus-targeted cells and organs would benefit delineation of the immune processes contributing to the establishment and endurance of the distinctive forms of infection and liver injury. The woodchuck model of HBV infection is uniquely positioned to advance knowledge in this area and it has been utilized, at least to some degree, for this purpose (Zhang et al., 2012; Meng et al., 2016). This is well examplified in studies on the oral TLR7 agonist GS-9620, which antiviral effects has been shown in woodchuck and chimpanzee models of CH type B, and its testing advanced to clinical trials suggesting that activated both innate and adaptive immunity contribute to antiviral response (Gane et al., 2015; Menne et al., 2015; Boni et al., 2018; Niu et al., 2018). Nevertheless, expression of the entire family of TLR genes in sequential forms of WHV infection and stages of coinciding hepatitis, as well as in healthy animals, has not yet been determined. The main goal of this study was to systematically determine the transcription profiles of TLRs 1 to 10 during the course of experimental infection and at the sites naturally targeted by hepadnavirus, i.e., liver and immune system, exemplified in this study by PBMC. For this purpose, serial liver biopsies and PBMC samples from woodchucks followed from the pre-infection period (healthy) throughout pre-acute (PreAH) and acute hepatitis (AH) to SLAH followed by SOI or CH, and from animals with long-term POI were investigated. We also aimed to recognize how the TLR expression profiles may differ between livers and primary hepatocytes isolated from these livers in different forms of infection and stages of hepatitis. This study was specifically focused on determination of the transcriptional activity of multiple TLR genes and generated wealth of information which should provide a direction and benefit further delineation of unique characteristics of the innate immune responses operating in successive stages and at different sites of hepadnaviral infection. This is particularly important since specific antibodies against the majority of woodchuck TLRs and affiliated molecules mediating the downstream effects of their activation or suppression remain unavailable.

## Materials and Methods

### Ethics Statement

All animal experiments and the animal maintenance protocols were performed in compliance with the Institutional Animal Care Committee at Memorial University, St. John’s, Newfoundland and Labrador, Canada that follows the guidelines and is accredited by the Canadian Council on Animal Care in Science. The approved protocol identification number is 13-159-M.

### Animals and Categories of WHV Infection and Hepatitis

Woodchucks (*Marmota monax*) were maintained in the Woodchuck Viral Hepatitis Research Facility at the Health Science Center, Memorial University, St. John’s, NL, Canada. Healthy animals were WHV-naive, as ascertained by negative results of serum testing for WHsAg and anti-WHc (Michalak et al., 1999). They also were negative for WHV DNA in serum and liver biopsies by high-sensitivity polymerase chain reaction (PCR)-based assays (Michalak et al., 1999, 2004). WHV-naïve woodchucks infected with WHV doses (≥>10^6^ DNA-ase digestion-protected virions) at one or 2-years of age developed AH accompanied by high levels of serum WHV DNA and WHsAg, as well as elevated biochemical indicators of liver injury, such as serum sorbitol dehydrogenase (SDH) (Gujar et al., 2013). If serum WHsAg cleared prior to 6 months post-infection, a diagnosis of SLAH was made. As indicated previously, SLAH is followed by the lifelong persistence of WHV DNA and anti-WHc reactivity in serum, while virus replication continues at low levels in the liver and the immune system (Michalak et al., 1999, 2004). This form of asymptomatic infection was designated as SOI (Mulrooney-Cousins and Michalak, 2015). If WHsAg persists in circulation for longer than 6 months, CH is diagnosed. CH normally coincides with high levels of serum WHV DNA (up to 10^10^–10^11^ vge/mL) and increased levels of enzymes indicative of liver injury. Woodchucks with CH at a high frequency (≥85%) develop HCC (Popper et al., 1981; Michalak, 1998). In the current study, 4 of 10 woodchucks with CH developed HCC. It should be mentioned that a non-tumorous part of hepatic tissue was used only for investigation. For the purpose of this study, PreAH refers to the phase of infection after inoculation with a high dose of WHV (≥10^6^ virions) but prior to establishment of AH. Regarding POI, woodchucks infected with WHV at doses of less than 10^3^ virions develop entirely asymptomatic (silent) infection that is accompanied by the lack of serological markers of infection, such as WHsAg and anti-WHc (Michalak et al., 2004; Mulrooney-Cousins and Michalak, 2015). However, WHV DNA is detectable in serum, PBMC and in the lymphatic (immune) system, and the virus with time may spread to the liver without induction of hepatitis, as reported (Michalak et al., 2004; Gujar and Michalak, 2009; Mulrooney-Cousins et al., 2014). The mean WHV levels in plasma, liver and PBMC of the animals with different form of infection or/and hepatitis investigated in this study were shown in Supplementary Table S1. The samples were collected at the time of acquisition of liver biopsy or autopsy tissue, which was usually obtained at 6–12 month intervals. In addition to immunovirological and biochemical markers measured in circulation, histological examination of liver biopsies obtained by laparotomy and at autopsy served to diagnose the status liver disease and its progression or resolution (Michalak and Lin, 1994; Michalak et al., 1999; Mulrooney-Cousins et al., 2014; Mulrooney-Cousins and Michalak, 2015).

### Samples Investigated

This study had three interconnected parts. In the first part, tissue samples from liver, spleen, bone marrow and PBMC collected at autopsy from 16 animals were analyzed. This study group included 4 healthy woodchucks (between 2 and 4 years of age), 7 with SLAH/SOI, and 5 with WHsAg-positive CH of which two finally developed HCC (Supplementary Table S2). Samples from animals with SLAH/SOI were collected from 10 to 36 months post-infection (m.p.i.) with WHV, while those from woodchucks with CH from 20 to 42 m.p.i. For the second part of the study, livers and hepatocytes isolated from these livers were examined. The autopsy livers and paired hepatocytes were obtained from 26 animals, including 5 healthy (between 2 and 4 years of age), 4 within the PreAH phase acquired before or at 4 weeks p.i. (w.p.i.), 8 with SLAH/SOI obtained at 12–48 m.p.i., 6 with CH collected from 18 to 48 m.p.i., and 3 with POI obtained between 12 and 66 m.p.i. (Table 1). In the third part, sequential liver biopsies procured from 28 woodchucks were analyzed for TLR 1–10 expression, including 20 samples from healthy, 4 from the PreAH stage obtained prior to or at 4 w.p.i., 8 from AH obtained between one and 6 m.p.i., 3 from SLAH/SOI acquired at 12–48 m.p.i., 12 from CH collected between 18 and 48 m.p.i., and 14 from POI taken between 12 and 66 m.p.i. Sixty-one liver biopsies in total were investigated (Table 1). Non-HCC liver tissue was investigated only. Finally, 51 sequential PBMC samples were collected from 4 woodchucks prior to and throughout the course of WHV infection (Table 1). Two of the woodchucks developed AH and subsequently resolved hepatitis and established SOI (i.e., SLAH/SOI), while two others progressed from AH to CH and finally developed HCC.

**Table 1:** Woodchuck samples from different forms of experimental WHV infection and stages of hepatitis examined in this study.

Sample type	Total animal number	Sample number according to category of infection or hepatitis						Total sample number
		Healthy	PreAH	AH	SLAH/SOI	CH	POI	
Autopsy liver	26	5	4	0	8	6	3	26
Hepatocytes	26	5	4	0	8	6	3	26
Liver biopsy	28	20	4	8	3	12	14	61
PBMC	4	11	8	18	6	8	0	51

*PreAH, pre-acute hepatitis; AH, acute hepatitis; SLAH/SOI, self-limited acute hepatitis followed by secondary occult infection; CH, chronic hepatitis; POI, primary occult infection*.

### Cell Isolations

Hepatocytes were isolated from autopsy livers by the two-step collagenase perfusion technique, as described in the previous works from this laboratory (Michalak et al., 1989; Michalak and Lin, 1994; Churchill and Michalak, 2004). Hepatocyte purity was consistently greater than 98% as determined by phase-contrast microscopy and confirmed by flow cytometry after staining for asialoglycoprotein receptor (ASGPR) and by RT-qPCR assays designed for detection of potential immune cell contaminations (Diao et al., 1998; Guy et al., 2010, 2011). PBMC were isolated by density gradient centrifugation, as reported (Gujar and Michalak, 2005). Their viability was determined by trypan blue dye exclusion and normally exceeded 98%. All cells were stepwise cooled and subsequently frozen. They were stored in liquid nitrogen prior to analysis.

### RNA Extraction and Transcription

Total RNA was isolated using TRIzol reagent (Invitrogen Life Technologies, Burlington, ON, Canada) from approximately 100 mg of tissue, 5 × 10^6^ hepatocytes and 0.5–1 × 10^7^ PBMC. Each series of RNA extractions were done in parallel with mock extraction using sterile water instead of test samples as a contamination control. In order to remove DNA potentially tainting samples, RNA aliquots were treated with amplification grade DNase I following the manufacturer’s protocol (Invitrogen). RNA concentration and purity were determined by an NanoDrop 2000 instrument (Thermo Scientific, Waltham, MA, United States), while RNA integrity by an Agilent 2100 Bioanalyzer (Agilent Technologies, Santa Clara, CA, United States). Following the analysis, an RNA Integrity Number (RIN) was generated for each RNA sample. Samples with RIN 8 or above were used in the study. If RIN was below 8, RNA extraction was repeated. Each RNA sample (2 μg) was transcribed to cDNA as reported (Pham et al., 2004).

### Amplification of Woodchuck TLR Sequences and Their Cloning

Toll-like receptor forward (sense) and reverse (anti-sense) oligonucleotide primers were designed to amplify woodchuck TLR1, TLR2, and TLR4 to TLR10. Primers for woodchuck TLR3 (GenBank accession number EU586552) were previously published (Zhang et al., 2009). Our primers were generated using aligned TLR nucleotide sequences from a variety of mammalian species deposited in the Entrez Nucleotide Database^1^. Complete protein-coding sequences (exons) were queried and aligned using Sequencher v4 software (Gene Codes Corporation, Ann Arbor, MI, United States). The consensus sequences were constructed and primers were designed to amplify TLR fragments in regions of the highest interspecies homology. These primers were used for the initial amplification of woodchuck TLR 1–10 by end-point polymerase chain reaction preceded by reverse transcription step (RT-PCR) using cDNA derived from spleens and livers of two healthy woodchucks. In the following step, PCR products were purified and cloned using a TOPO TA cloning system (Invitrogen). Mini-scale preparations from individual clones were generated, DNA isolated and subjected to restriction enzyme digestion. The inserts of interest carrying exon DNA sequence for each of the woodchuck TLR genes were bidirectional sequenced to confirm the presence of TLR sequence. Then, large stocks of plasmids containing confirmed woodchuck TLR sequences were produced.

### Woodchuck-Specific TLR 1–10 Real-Time RT-qPCR Assays

Toll-like receptor primers used for the initial amplification of woodchuck TLR 1–10 fragments were redesigned, if required, based on the confirmed woodchuck sequences generated to be 100% woodchuck compatible. The final primer sequences used in this study are listed in Table 2. Each TLR primer pair underwent series of experiments determining optimal conditions for real-time (quantitative) RT-PCR (i.e., RT-qPCR) amplification. Each individual reaction included the cDNA equivalent of 50 ng of total RNA tested and 5 pmol of each respective primer. Reactions were performed using SsoFast EvaGreen Supermix (Bio-Rad Laboratories, Mississauga, ON, Canada) in a 96-well plate format and the Light Cycler 480 System (Roche Diagnostics, Mannheim, Germany). The sensitivity of detection for each TLR was determined using serial 10-fold dilutions of plasmid carrying respective woodchuck TLR sequence insert. All TLR primer pairs were able to detect at least 100 copies of a given TLR per reaction. TLR1, TLR2, TLR6, TLR9, and TLR10 were detectable at a limit of detection (LOD) of 10 copies/reaction (Table 2). Amplification of serial dilutions of known plasmid concentrations allowed for the generation of a standard curve for absolute quantification of a given TLR expression. Samples with *C*t values that fell within the standard curve range were assigned a copy number for the TLR tested.

**Table 2:** Woodchuck TLR 1–10 and housekeeping gene primer sequences used for quantitative expression analyses.

Gene	Primer sequence (5′–3′ orientation)		Length (bp)	Predicted amplicon size (bp)	Sensitivity (copies/reaction)
TLR-1	+	CAT TTG ATG CCC TGC CTA TAT G	22	435	10
	–	TAT GCC AAA CCA GCT GGA GGA T	22		
TLR-2	+	TGA CTC TCC CTC CCA C	16	235	10
	–	GTC GTA GCA GAT GTC CC	17		
TLR-3	+	AGG GAC TTT GAG GCA GGT GT	20	230	100
	–	CGC AAA CAG AGT GCA TGG T	19		
TLR-4	+	AAG GTT TCC ATA AAA GCC G	19	193	100
	–	AGT AGG CGG TAC AAC TC	17		
TLR-5	+	GCC TTG AAG CCT TCA GTT ATG C	22	76	100
	–	CCA ACC ACC ACC ATG ATG AG	20		
TLR-6	+	GCC CAA ACC TGT GGA ATA TCT CA	23	424	10
	–	CAA AGA ATT CCA GCT AAC ATC CA	23		
TLR-7	+	GCC TGT TCT GTA AAG G	16	471	100
	–	ACT CCC GGA ATG ATT G	16		
TLR-8	+	CAC ATC CCA AAC TTT CTA TGA TG	23	100	100
	–	CTC TTC AAG GTG GTA GCG C	19		
TLR-9	+	TGG TAC TGC TTC CAC CT	17	358	10
	–	ACA CCA CGA CAT CCT T	16		
TLR-10	+	GAT GGT CAG ATT CAT ACA TCT G	22	124	10
	–	ATG ATG GCC ACA ATG GTG AC	20		
HPRT	+	TGA CAC TGG CAA AAC AAT GCA	21	96	10
	–	GGT CCT TTT CAC CAG CAA GCT	21		
β-actin	+	ATC ATG TTT GGG ACC TTC AA	20	350	10
	–	CAT CTC TTG GTC GAA GTC CA	20		

The layout for each 96-well plate was designed to include all appropriate controls in addition to test samples, i.e., (1) All components of the amplification reaction except the cDNA template (no template control, NTC); (2) Sterile water instead of RNA template when reverse transcribing RNA to cDNA (negative RT, mock); (3) Internal controls from livers and spleens of two healthy woodchucks carrying known copy numbers of each TLR 1–10, and (4) Serial dilutions 1 × 10^1^ to 1 × 10^6^ copies of the appropriate recombinant TLR fragment as quantitative standards. The NTC served for excluding potential contamination in the PCR reagents, while the mock controlled for any potential contamination carryover from RT reagents. Amplifications of all test and control samples were done in triplicate.

### Determination of TLRs’ Expression

Expression of woodchuck β-actin was evaluated as housekeeping gene in the first phase of the study when profiles of TLRs’ expression in woodchuck autopsy samples were compared (Supplementary Figure S1). However, β-actin was found to be unreliable when comparing TLRs transcription between livers and hepatocytes (Supplementary Figure S2). Therefore, TLR expression was subsequently normalized to that of hypoxanthine-guanine phosphoribosyl transferase (HPRT). HPRT was chosen based on the previous studies analyzing the expression of multiple housekeeping genes which concluded that HPRT is one of the most stably transcribed genes in human and rat livers and hepatocytes (Nishimura and Naito, 2005; Chen et al., 2006; Tsaur et al., 2013). Thus, the absolute TLR expression values were divided by the absolute expression value of HPRT or β-actin.

### DNA Extraction and WHV Quantification

DNA from 100 to 400 μL of serum or plasma and from liver, spleen, bone marrow, and PBMC was extracted by the proteinase K-phenol-chloroform method (Michalak et al., 1999; Mulrooney-Cousins et al., 2014). Considering serum or plasma samples, only samples collected at the time of acquisition of liver tissue at biopsy or autopsy or PBMC were examined. WHV DNA was quantified by real-time PCR (sensitivity, 10–100 vge/mL) using DNA equivalent to 25 μL of plasma or 400 ng of total DNA from cells or tissues, and WHV C and X gene primers (Mulrooney-Cousins et al., 2014; Chauhan et al., 2017). When applicable, WHV DNA was semi-quantified by direct and, if negative, nested PCR/nucleic acid hybridization (NAH) using primers and conditions reported (Michalak et al., 1999; Coffin et al., 2004; Mulrooney-Cousins et al., 2014). Mock extractions and respective nucleic acid preparations from WHV-positive and WHV-negative woodchuck livers or PBMC were included as controls. NAH analysis of PCR products was routinely performed to verify the specificity of virus detection and the validity of controls (Michalak et al., 1999; Coffin et al., 2004; Mulrooney-Cousins et al., 2014).

### Statistical Analysis

Results were analyzed by paired or unpaired Student’s-*t* test, where applicable, using GraphPad Prism software (Graph Pad Software Inc., San Diego, CA, United States). A paired *t*-test was used when analyzing TLR 1–10 mean expression values in livers and hepatocytes derived from those livers. Differences between experimental conditions were considered to be significant when two-sided *P*-value from analysis of mean expression levels was equal or lower than 0.05. Data bars marked with ^∗^ were significant at *P* < 0.05, ^∗∗^ at *P* ≤ 0.01, ^∗∗∗^ at *P* ≤ 0.001, and ^∗∗∗∗^ at *P* ≤ 0.0001.

### Accession Numbers

Woodchuck TLR partial gene sequences identified in the current study were deposited in GenBank. Their accession numbers are KY468972 to KY468981 for TLR1 to TLR10, respectively.

## Results

### Woodchuck TLRs 1–10 Sequence Compatibility With Human TLR Sequences

Alignment of amino acid (aa) sequences predicted from woodchuck TLR 1–10 nucleotide sequences identified in this study with respective aa sequences of human TLRs 1–10 showed that, except for TLR1 and TLR6, the sequences coded for the Toll-interleukin 1 receptor (TIR) domain of the TLR proteins. Hence, woodchuck TLR3 (aa 790–870), TLR4 (aa 710–810), and TLR5 (aa 770–800) aligned with a region located within the TIR domain of human TLR3 (GenBank accession number ABC86910), TLR4 (GenBank AAF05316) and TLR5 (GenBank AAI09119), respectively. Woodchuck TLR8 (aa 880–910) and TLR9 (aa 840–1030) spanned the cytoplasmic region located upstream from the TIR domain and overlapped with the TIR domain of human TLR8 (GenBank AAZ95441) and TLR9 (GenBank AAZ95521). Further, woodchuck TLR2 (aa 540–670), TLR7 (aa 790–1030) and TLR10 (aa 520–693) corresponded to the extracellular, transmembrane and TIR domains of human TLR2 (GenBank AAH33756), TLR7 (GenBank AAZ99026) and TLR10 (GenBank AAY78491), respectively. In contrast, TLR1 (aa 120–270) and TLR6 (aa 280–420) were homologous to the cytoplasmic domain of human TLR1 (GenBank AAH33756) and TLR6 (GenBank BAA78631). This was expected, since knowing that the TIR domains of TLR1 and TLR6 share high sequence homology, making them undesirable for specific priming (Plain et al., 2010), we designed primers for the regions sharing the least homology. This allowed for specific quantification of woodchuck TLR1 and TLR6 expression in our study.

We acknowledge that when this study was in progress, partial sequences for woodchuck TLR3 (GenBank EU586552.1), TLR4 (GenBank EU586553.1), TLR7 (GenBank EU586554.1), TLR8 (GenBank EU586555.1), and TLR9 (GenBank EU586556.1) were reported (Zhang et al., 2009). Later, a partial sequence of woodchuck TLR2 (GenBank HQ446273.1) and the complete sequence coding for woodchuck TLR7 (GenBank KT013099.1) also became available. Nonetheless, when these sequences were compared to the woodchuck TLR gene fragments identified in the current study all, but TLR3 and TLR7, were either fully or partially unique, and have not yet been previously reported. For the GenBank accession numbers of the woodchuck TLR sequences identified in this study, see section “Materials and Methods”.

### TLRs 1–10 Expression in Livers, Lymphatic Tissues and PBMC of Healthy Woodchucks

**FIGURE 1:**
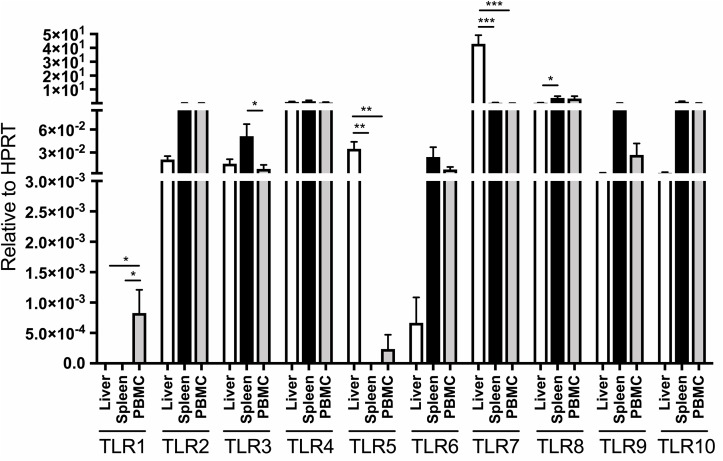
Distribution of TLR 1–10 expression in liver, spleen, bone marrow and PBMC obtained from four healthy adult woodchucks. Autopsy tissues and PBMC were evaluated for transcription of individual woodchuck TLRs by qRT-PCR assays as outlined in section “Materials and Methods”. The TLR expression was normalized to expression of woodchuck HPRT. The results are presented as relative mean values ± SEM. Statistically significant differences between data bars are marked with ^∗^, ^∗∗^ or ^∗∗∗^ as explained in section “Materials and Methods”. TLR1 was not detectable in liver, spleen and bone marrow but was at low copy numbers in PBMC. HPRT was not detectable in bone marrow samples and therefore is not shown.

Liver, spleen, bone marrow and PBMC collected at autopsy from four healthy, WHV-naïve adult woodchucks (Supplementary Table S2) were evaluated for expression of TLRs 1–10 by assays applying real-time RT-qPCR, as detailed in section “Materials and Methods”. To enable comparisons between tissues, expression of individual TLRs was normalized to the expression of woodchuck HPRT and β-actin (Figure 1 and Supplementary Figure S3, respectively). Both house-keeping transcripts were detectable by RT-qPCR with a LOD of 10 copies per reaction (Table 2).

The expression of individual TLRs was variable depending on the class of TLR and organ investigated, and was often influenced by housekeeping gene used for normalization of their expression. When expression of individual TLRs were adjusted to that of HPRT, liver TLR5 and TLR7 were transcribed at significantly greater levels than those in PBMC and spleen (Figure 1). In addition, TLR8 was expressed at a significantly higher level in spleen than liver but not in PBMC, while TLR1 transcription remained significantly higher in PBMC than in liver or spleen (Figure 1). Notably, bone marrow samples did not express detectable levels of HPRT. For comparison, when related to β-actin expression, normal liver tissue displayed significantly greater mean levels of TLR3, TLR4, TLR5, and TLR7 than spleen, bone marrow or PBMC, while hepatic mean TLR2 transcription was meaningfully higher than that in spleen and PBMC (Supplementary Figure S3). TLR6 expression was significantly higher in PBMC than bone marrow, but displayed at comparable levels in bone marrow, spleen and liver tissues. Further, TLR8 was ubiquitously expressed in the tissues examined and PBMC. Only TLR10 was transcribed in the spleen at significantly higher level than in liver or bone marrow (*P* < 0.05), but at similar mean level as in PBMC when adjusted to β-actin expression (Supplementary Figure S3). TLR1 expression was found only in PBMC, while that of TLR5 only in the liver but not in other samples examined (Supplementary Figure S3).

### Comparative Analysis of TLRs 1–10 Expression in Woodchucks With Resolved Acute Hepatitis and Chronic Hepatitis

Since virological and immunological features, as well as a degree of hepatocellular damage and liver inflammation significantly vary between different forms of WHV infection, TLRs 1–10 expression was assessed in autopsy liver, spleen, bone marrow, and PBMC from two opposed forms of WHV-induced liver injury. Thus, samples were obtained from animals with SLAH (abbreviated also as SL) or CH (Supplementary Table S2). SLAH is naturally followed by lifelong SOI (i.e., SLAH/SOI) and accompanied by histologically evident intermitted minimal and, rarely, mild hepatitis (Michalak et al., 1999). The mean WHV levels in livers and PBMC of SLAH/SOI animals investigated in this part of the study (*n* = 7) were 1.0 × 10^3^ (SEM ± 5.4 × 10^2^) and 2.0 × 10^3^ (SEM ± 1.1 × 10^2^) vge/μg DNA, respectively. Woodchucks with CH demonstrated histologically evident mild to severe chronic liver inflammation which coincided with continuous presence of WHsAg and high loads of WHV DNA in serum (*n* = 5). The mean WHV level in livers of these animals was 2.8 × 10^7^ (SEM ± 2.3 × 10^7^), while that in PBMC was 4.2 × 10^1^ (SEM ± 2.4 × 10^1^) vge/μg DNA. The expression of TLRs was compared to that in the respective tissues or PBMC from healthy animals (*n* = 4) (Supplementary Table S2). Since HPRT was not transcribed at measurable levels in woodchuck bone marrow, β-actin was used for normalization of TLRs’ expression for this part of the study (Supplementary Figure S4).

Considering the liver, a significant downregulation in the expression of TLR5 in animals with CH comparing to that in animals with SLAH/SOI (*P* < 0.05) and healthy woodchucks (*P* < 0.001) was found. Furthermore, the mean expression of TLR9 in hepatic tissue of woodchucks with CH was significantly greater than that in healthy animals (*P* < 0.05) and tended to be higher, but not at a statistically significant level, than that in animals with SLAH/SOI. In addition, TLR9 mean expression showed a similar trend as TLR6 in CH liver. The transcription levels of all other TLRs were not meaningfully different between these three study groups (Supplementary Figure S4).

Among lymphoid tissues and PBMC, the spleen most frequently demonstrated upregulated expression of individual TLRs which coincided with serologically evident chronic hepatitis (Supplementary Figure S4). Thus, the expression of TLR2 was significantly augmented in animals with CH comparing to those with SLAH/SOI (*P* < 0.05) or healthy woodchucks (*P* ≤ 0.001). Moreover, TLR4 (*P* ≤ 0.001), TLR8 (*P* < 0.05), and TLR9 (*P* < 0.05) were transcribed at significantly greater levels in spleens of woodchucks with CH than in those of healthy animals. Also, it of notice that TLR8, TLR9, and TLR10 showed the same expression trends CH spleens. In addition, the TLR mean expression levels tended to be higher in animals with SLAH/SOI comparing to those in healthy, but without achieving statistical significance.

Considering PBMC, the level of TLR1 expression was significantly higher in SLAH/SOI comparing to that in either CH or healthy woodchucks (*P* < 0.05). Also, the expression of TLR3 was greater in SLAH/SOI than CH (*P* < 0.05), but not meaningfully different from that in health animals (Supplementary Figure S4). There were no statistically significant differences between the groups in the mean expression levels of TLRs 1–10 in samples of bone marrow.

Overall, comparative analysis of the TLR’s expression in liver and three different locations within the immune system from SLAH/SOI, CH and healthy animals showed distinct profiles regarding transcription of individual TLRs when normalized to β-actin. The differences were most pronounces in liver and spleen, less in PBMC, and not apparent in bone marrow.

### Expression of TLRs 2–10 in Livers and Isolated Hepatocytes

It became apparent that determination of expression of housekeeping genes was important to ensure that they were detectable and at approximately similar levels across the organs and cells examined to facilitate comparative analysis of TLR expression. Thus, the preceding evaluations included assessment of expression of woodchuck β-actin and HPRT in livers (*n* = 26) and hepatocytes isolated from these livers (*n* = 26) in healthy (*n* = 5) and WHV-infected (*n* = 21) woodchucks. The results showed that β-actin was expressed at significantly higher mean levels in liver tissue samples (*P* < 0.05) than in isolated hepatocytes (Supplementary Figure S2A). In contrast, HPRT was transcribed at comparable levels in both hepatic tissue (*n* = 87) and hepatocytes (*n* = 26). It is of note that transcription of HPRT was significantly greater in PBMC (*n* = 51) than in liver tissue samples (*n* = 87) (*P* < 0.0001) or hepatocytes (*n* = 26) (*P* < 0.0001) (Supplementary Figure S2B). Therefore, TLR expression in relation to HPRT in livers and hepatocytes were compared to each other in all forms of WHV infection and hepatitis, while that in PBMC to livers and hepatocytes from healthy woodchucks only.

Since liver parenchyma is comprised by 85% of hepatocytes, we aimed to assess whether primary hepatocytes express similar profiles of TLR 1–10 expression as the livers during WHV infection. For this part of the study, paired liver tissue samples and purified hepatocytes prepared from these livers were obtained from 26 animals, including 5 healthy woodchucks (four male and one female), 4 animals infected with WHV but prior to diagnosis of AH (i.e., PreAH) (three male and one female), 8 woodchucks which resolved AH and established SOI (i.e., SLAH/SOI) (two male and six female), 6 during CH (four male and two female), and 3 in the course of POI (two male and one female) (Table 1).

**FIGURE 2:**
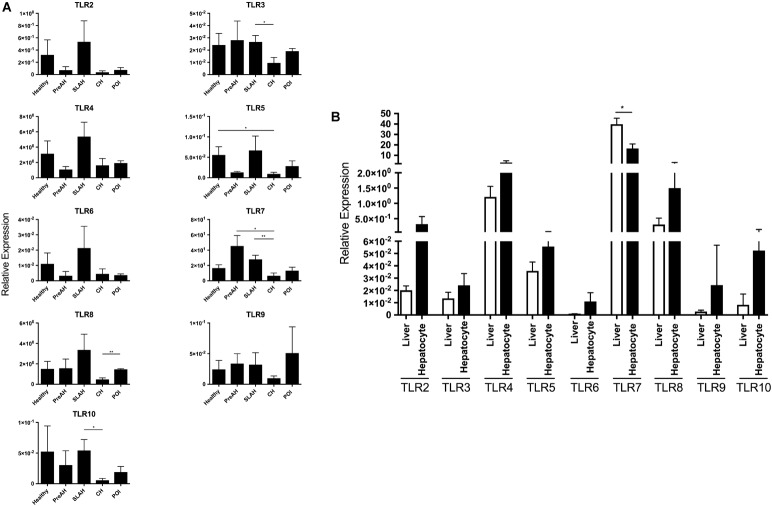
TLR 2–10 expression profiles in livers and hepatocytes isolated from these livers obtained from healthy and WHV-infected woodchucks. Transcription levels of TLR 2–10 in livers and hepatocytes were acquired from healthy animals (*n* = 5) and woodchucks with pre-acute phase of hepatitis (PreAH; *n* = 4), after recovery from acute hepatitis during secondary occult infection (SLAH; *n* = 8), chronic hepatitis (CH; *n* = 6) or in the course of primary occult infection (POI) (*n* = 3). TLRs’ expression was normalized against HPRT expression and presented as relative mean values ± SEM. **(A)** Expression of TLRs 2–10 in hepatocytes obtained from distinct forms of WHV infection and stages of hepatitis. **(B)** Expression levels of each individual TLR in livers of WHV-naïve, healthy animals and in primary hepatocytes derived from these livers. Differences between data bars are marked as indicated in section “Materials and Methods”. TLR1 transcription was evaluated but not detected.

Considering hepatocytes alone, when compared to hepatocytes from CH, hepatocytes from SLAH/SOI showed upregulated mean expression of TLR3 (*P* < 0.05), TLR7 (*P* < 0.01) and TLR10 (*P* < 0.05), while those from healthy animals demonstrated significantly higher levels of TLR5 (*P* < 0.05) (Figure 2A). Hepatocytes from woodchucks with CH also displayed significantly lower mean expression levels of TLR7 and TLR8 when compared to animals with PreAH (*P* < 0.01) and POI (*P* < 0.01), respectively. In addition, the mean expression of TLRs 2–4, 6, and 8–10 in hepatocytes from healthy animals and TLRs 2, 4, 6, 8, and 9 in hepatocytes from SLAH/SOI tended to be higher than in hepatocytes from CH, but these differences were not statistically significant (Figure 2A). Overall, hepatocytes from animals with CH were characterized by significant downregulation or trend toward suppression of transcription of almost all TLRs when compared to hepatocytes from other forms of WHV infection and from healthy animals.

Transcription levels of TLRs 1–10 were also compared between livers and hepatocytes isolated from these livers. Considering healthy animals, only the mean level of TLR7 expression was significantly downregulated in hepatocytes comparing to that in the total liver tissue (*P* < 0.01) (Figure 2B). However, although without reaching level of statistical significance, mean expression levels of all other TLRs were upregulated in hepatocytes when compared to normal liver tissue. This indicated that the TLR expression profile was modified in hepatocytes following isolation, particularly that of TLR7. This result should be taken under consideration when interpreting data on TLRs’ expression from experiments utilizing primary hepatocytes.

**FIGURE 3:**
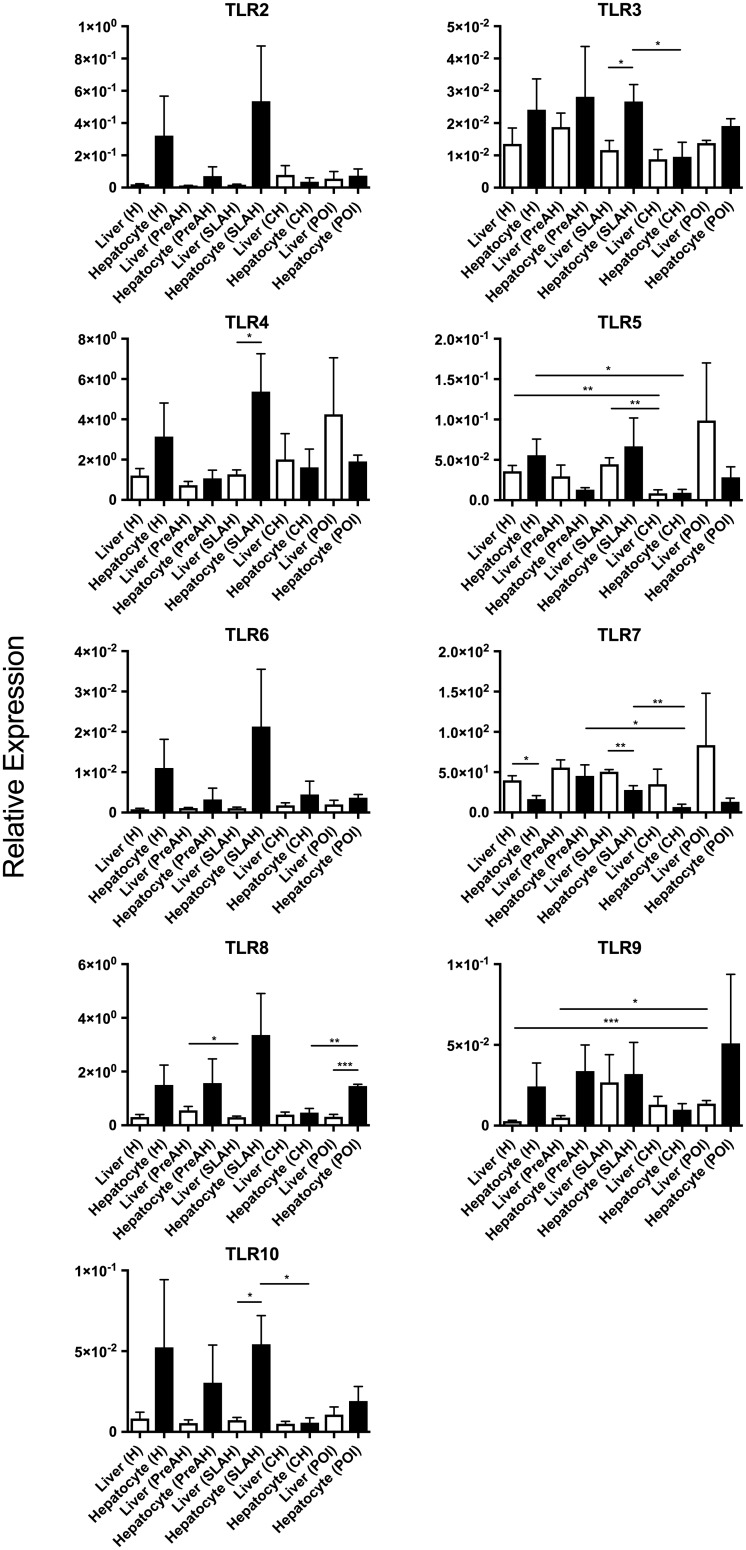
Comparative expression of individual TLRs in livers or hepatocytes isolated from these liver within each group with the same form of infection or hepatitis and in livers or hepatocytes from a particular group across all study groups. Differences between data bars reflecting relative mean expression values ± SEM are marked as indicated in section “Materials and Methods”.

Animals with SLAH/SOI also demonstrated a significantly greater mean expression level of TLR7 in livers than hepatocytes (*P* < 0.01) (Figure 3). Conversely, livers from woodchucks with SLAH/SOI displayed significantly decreased mean expression of TLR3 (*P* < 0.05), TLR4 (*P* < 0.05), and TLR10 (*P* < 0.05) when compared to hepatocytes derived from these livers. Furthermore, TLR8 expression was also found at significantly lower mean level in livers than hepatocytes (*P* < 0.001) in animals with POI (Figure 3). There were no significant differences in mean expression of TLR2, TLR6, and TLR9 between livers and hepatocytes from healthy or WHV-infected woodchucks (Figure 3). However, there was apparent consistency in augmented, but not statistically different, expression of TLR2, TLR4, TLR6, and TLR 8–10 in hepatocytes over livers from SLAH/SOI and healthy woodchucks. This extended to TLR6 and TLR 8–10 for hepatocytes versus livers from the PreAH phase. Finally, TLR1 was not detected in any of the liver and hepatocyte samples. In general, the results showed that while healthy animals and woodchucks with PreAH, CH or POI showed a statistically significant difference in expression of only one particular TLR between livers and hepatocytes derived from these livers, animals with SLAH/SOI demonstrated highly pronounced differences in this regard.

### Expression Profiles of TLRs 2–10 in Sequential Liver Biopsies Acquired Prior to and During Experimental WHV Infection

**FIGURE 4:**
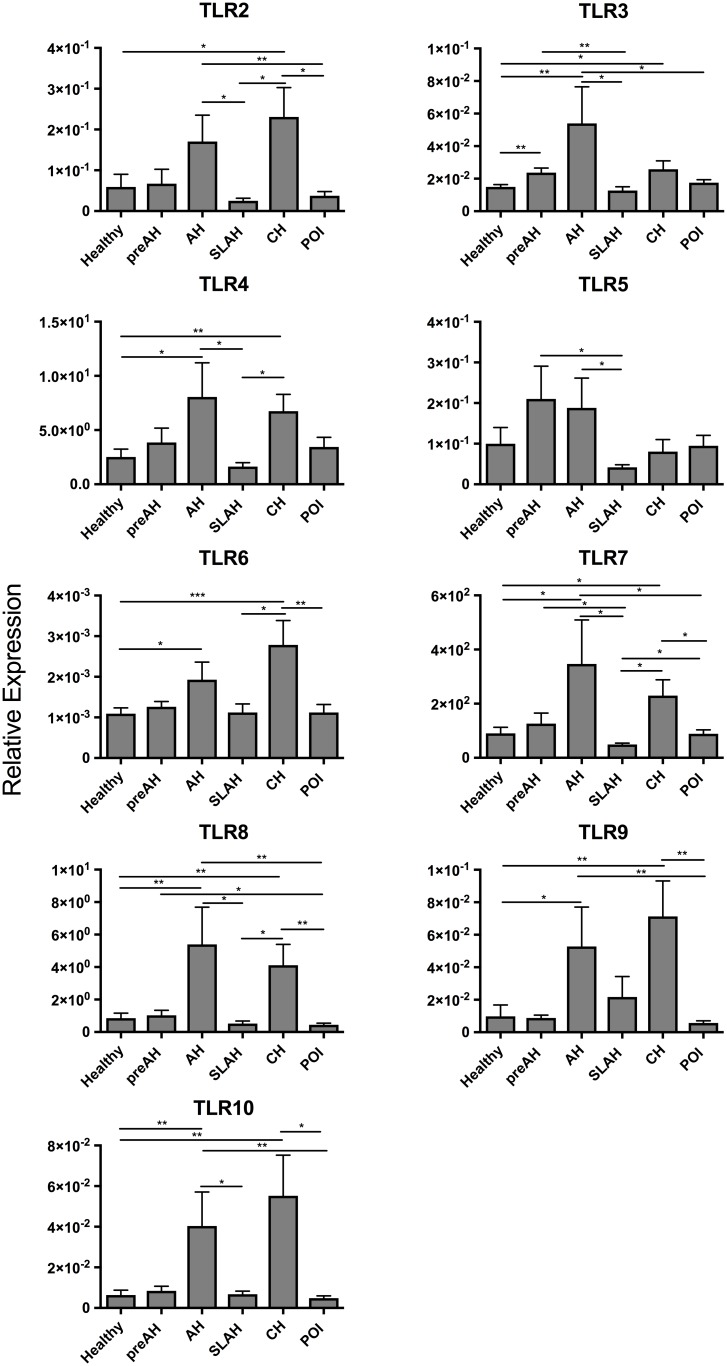
Transcription levels of TLR 2–10 in sequential liver biopsies and liver tissue samples collected at autopsy from woodchucks with different forms of WHV infection and hepatitis. Liver samples were obtained from healthy woodchucks (*n* = 25) and animals with pre-acute phase of hepatitis (PreAH; *n* = 8), during acute hepatitis (AH; *n* = 8), following resolution of acute hepatitis (SLAH; *n* = 11), during chronic hepatitis (CH; *n* = 18) or in the course of primary occult infection (POI; *n* = 17). TLR1 was not detectable. For more details, see the legend to Figure 2 and section “Materials and Methods”.

Liver tissue samples acquired at biopsies of healthy animals, throughout the course of WHV infection induced in these animals and during autopsy, at the end of follow-up, were investigated. There were in total 25 liver tissue samples collected from healthy woodchucks, 8 from the PreAH phase, 8 from AH, 11 from SLAH/SOI, 18 from CH, and 17 from POI (Table 1). When compared to the healthy, pre-infection stage, the PreAH phase was characterized by significantly greater expression of TLR3 (*P* < 0.01) (Figure 4). PreAH livers also showed significantly higher transcription levels of TLR3 (*P* < 0.01), TLR5 (*P* < 0.05) and TLR7 (*P* < 0.05) when compared to the SLAH/SOI phase, while TLR8 (*P* < 0.05) was upregulated when compared to livers from animals with POI. In addition, although not significantly, PreAH livers showed increased expression of TLR 4–7 comparing to livers of healthy animals, TLR2, TLR4, TLR6, and TLR8 when compared to livers from the SLAH/SOI phase, and TLR 2–5 and TLR 7–8 when compared to livers from POI. In contrast, TLR9 appeared to be downregulated in PreAH livers versus healthy livers and those from animals with SLAH/SOI, while upregulated in SLAH/SOI versus POI. Analysis of liver biopsies from the AH stage showed significant upregulation of several TLR genes, specifically, TLR3 (*P* < 0.01), TLR4 (*P* = 0.01), TLR6 (*P* < 0.05), TLR7 (*P* = 0.01), TLR8 (*P* < 0.01), TLR9 (*P* < 0.05), and TLR10 (*P* < 0.01) when compared to their expression in healthy liver tissue (Figure 4). Upregulated transcriptional levels of hepatic TLRs were also found when comparing liver samples from AH with both SLAH/SOI and POI forms of the infection. Thus, the AH stage livers had significantly higher transcript levels of TLRs 2–5, 7, 8, and 10 (for all *P* < 0.05) compared to SLAH/SOI. AH livers also showed higher expression of TLR2 (*P* < 0.05), TLR3 (*P* < 0.05), TLR7 (*P* < 0.05), TLR8 (*P* < 0.01), TLR9 (*P* < 0.01), and TLR10 (*P* < 0.01) when compared to those from POI. Furthermore, livers from woodchucks with CH transcribed significantly more TLR2 (*P* < 0.05), TLR3 (*P* < 0.05), TLR4 (*P* < 0.01), TLR6 (*P* < 0.001), TLR7 (*P* < 0.05), TLR8 (*P* < 0.01), TLR9 (*P* < 0.01), and TLR10 (*P* < 0.01) than livers of healthy animals. Liver biopsies from woodchucks with CH also had significantly greater transcriptional levels of TLR2 (*P* < 0.05), TLR4 (*P* < 0.05), TLR6 (*P* < 0.05), TLR7 (*P* < 0.05), and TLR8 (*P* < 0.05) in relation to the SLAH/SOI stage. Also, TLR2 (*P* < 0.05), TLR6 (*P* < 0.01), TLR7 (*P* < 0.05), TLR8 (*P* < 0.01), TLR9 (*P* < 0.01), and TLR10 (*P* < 0.05) were transcribed at significantly higher levels in the CH phase when compared to the liver tissue obtained during POI (Figure 4). Finally, livers from POI had significantly higher expression levels of TLR7 (*P* < 0.05) when compared to those from the SLAH/SOI phase. TLR1 was undetectable in any of liver biopsies examined. In summary, there was a trend toward an overall increase in hepatic TLR expression when comparing liver samples from PreAH, AH, and CH stages to those from animals with SLAH/SOI or POI and healthy woodchucks.

The observed kinetics of TLRs 1–10 expression in sequential liver biopsy and PBMC samples acquired from two male woodchucks before inoculation with WHV and during progression of infection from the pre-acute phase to CH are shown in Supplementary Figure S5. These data illustrate individual variations in the TLRs’ expression prior to and during comparable stages of WHV infection. Thus, TLR2, TLR4, TLR6, TLR9, and TLR 10 expression was not upregulated in AH and/or CH comparing to healthy liver in woodchuck #1, while upregulated TLR 2–9 expression during AH, but not during CH, was found in woodchuck #2. It is of note that the same two animals (plus others) also provided data included in Figure 4. Together, the results indicate that although individual animals may display variability in expression of certain TLRs (Supplementary Figure S5), they also reveal that most of animal groups with different forms of infection/hepatitis significantly differed in their TLR expression patterns (Figure 4).

### Expression of TLRs 1–10 in PBMC of Healthy Animals and During Different Forms of WHV Infection and Hepatitis

**FIGURE 5:**
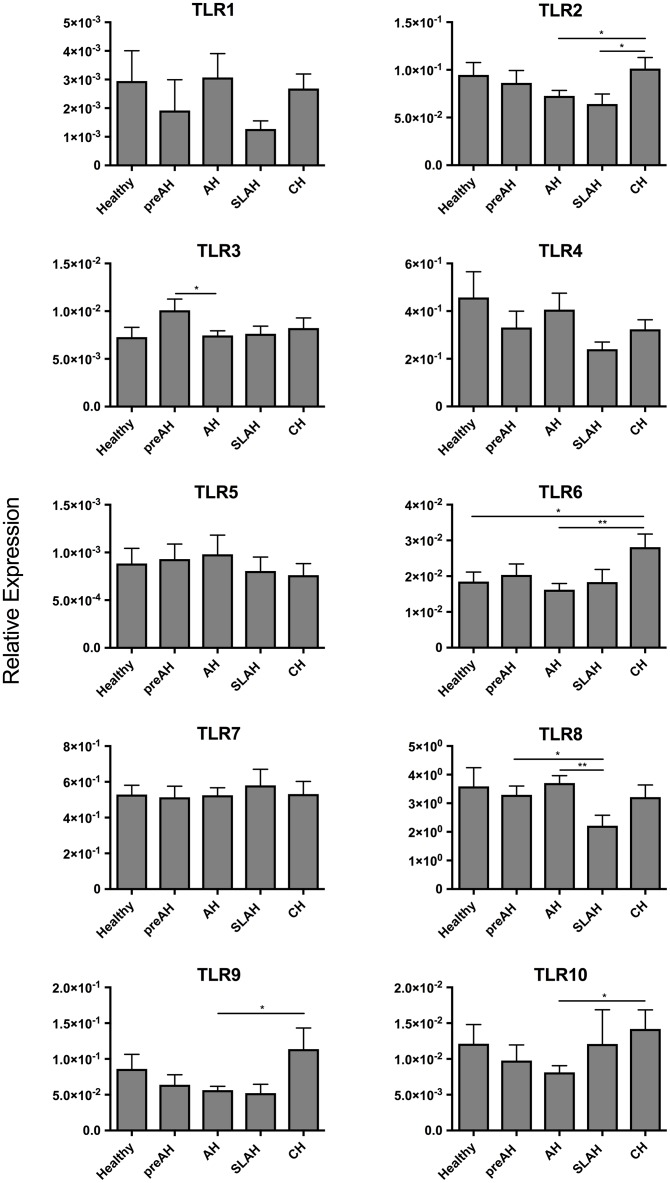
Profiles of TLR 1–10 expression in sequential PBMC samples from healthy woodchucks and those with different forms of WHV infection and hepatitis. Transcription levels of TLR 1–10 in PBMC acquired from healthy animals (*n* = 11) and in different phases of WHV infection, including PreAH (*n* = 8), AH (*n* = 18), SLAH/SOI (*n* = 6) and CH (*n* = 8). For details, see the legend to Figure 3 and section “Materials and Methods”.

Sequential PBMC samples collected from healthy woodchucks and during progression of WHV infection were evaluated for the expression levels of TLRs 1–10 (Table 1). PBMC from woodchucks with CH showed upregulated expression of several TLRs when compared to the PBMC from healthy animals and those from other study groups (Figure 5). When comparing to PBMC from healthy woodchucks and those from animals with SLAH/SOI, PBMC from CH had significantly higher expression levels of TLR2 (*P* < 0.05) and TLR6 (*P* < 0.05). In addition, they transcribed significantly higher levels of TLR2 (*P* < 0.05), TLR6 (*P* < 0.01), TLR9 (*P* = 0.01), and TLR10 (*P* = 0.01) in comparison to PBMC from AH (Figure 5). TLR8 was significantly downregulated in the cells from SLAH/SOI when compared to PBMC from the PreAH phase (*P* < 0.05) and AH (*P* < 0.01). Whereas TLR3 had higher expression levels in PBMC from the PreAH stage (*P* < 0.05) when compared to the cells from AH. In contrast to liver and hepatocyte samples, PBMC readily expressed TLR1, but the expression levels did not significantly vary between healthy and woodchucks with different forms of infection and stages of hepatitis. Overall, the dynamic of expression of individual TLRs was noticeably less pronounced in PBMC than in liver biopsies obtained from the same stages of WHV infection and hepatitis, although there was some compatibility observed. For example, expression of TLR2 was significantly greater during CH than AH in both liver biopsies and PBMC. In addition, significantly higher expression of TLR6 in CH than in the period prior to infection, TLR8 in AH than in the SLAH/SOI phase, and TLR10 in CH than in AH were apparent in both PBMC and liver biopsies. Nonetheless, the study uncovered that analysis of TLRs’ expression in PBMC does not reflect reliably the liver profiles of TLRs’ transcription. An example of TLRs 1–10 expression kinetics in sequential PBMC and liver biopsy samples collected during progression of WHV infection to CH is shown in Supplementary Figure S5.

## Discussion

The expression of TLRs 1–10 genes in livers, primary hepatocytes and PBMC of healthy woodchucks and those with different forms of experimental WHV infection and stages of hepatitis was investigated in this study. To accomplish this, woodchuck TLR 1–10 gene exon sequences were identified and primers specific for TLRs 1–2 and 4–10 were designed. These primers, together with woodchuck TLR3 primer pair previously reported (Zhang et al., 2009), were optimized for specific amplification of target sequences in RT-qPCR assays. To provide absolute quantification, each TLR gene sequence was cloned and serial dilutions of recombinant DNA sequences served as absolute quantification standards. This facilitated enumeration of TLRs’ expression with high accuracy and sensitivity. Among the most interesting findings of the study was identification that: (1) certain profiles of TLRs’ expression tended to coincide with particular forms of WHV infection; (2) analysis of the TLRs transcription in hepatocytes provided more distinct association with infection form than that of total liver tissue, as in case of CH and SLAH/SOI, and (3) the profiles of TLRs’ expression in PBMC rather poorly correlated with those of the liver and hepatocytes observed in different forms of infection. In addition, this study for the first time established baseline TLRs’ expression in healthy woodchucks and delineated the profiles of TLRs 1–10 transcription during progression of infection in the same animal from the healthy state to CH or SLAH/SOI.

Studies on expression of TLR genes during HBV infection were almost entirely limited to investigations of PBMC, as acquisition of liver biopsies, particularly sequential samples, from patients during progression of hepatitis B is essentially not feasible (Akbal et al., 2017). In this context, woodchucks experimentally infected with WHV offer an unmatched research advantage by enabling collection of serial liver and PBMC samples at experimentally defined time points post-infection when the status of infection and liver disease can be accurately determined. In the current study, samples collected from WHV-infected woodchucks were representative of all forms of infection and stages of hepatitis, as the data on serological (i.e., immunovirological) markers, viral loads (Supplementary Table S1) and liver histology attested.

Investigations of TLR transcription in healthy humans showed that the liver was one of the organs transcribing TLRs at the lowest levels (Zarember and Godowski, 2002; Nishimura and Naito, 2005). This decreased basal hepatic TLRs’ expression has been proposed to contribute to high immune tolerance to intestinal antigens to which this organ is repetitively exposed (Mencin et al., 2009). In contrast, human spleen and PBMC have the highest expression levels of TLRs 1–10 among organs tested (Zarember and Godowski, 2002). One study comparing human PBMC to hepatic tissue has shown that all TLRs were transcribed at a higher level in PBMC than in the liver, except TLR3, when the levels were adjusted to house-keeping genes including β-actin (Zarember and Godowski, 2002). In the current study, mean TLR3 mRNA levels in normal woodchuck livers also was greater than that in PBMC when related to the β-actin expression (see Supplementary Figure S3). Nonetheless, this difference was not observed when TLR3 expression was adjusted to HPRT expression in the same locations (see Figure 1). The same applied to expression of TLR2 and TLR4 after adjustment to HPRT (Figure 1) instead of β-actin for which liver showed significantly upregulated expression of both TLRs when compared to lymphoid tissues (Supplementary Figure S3). Notably, mean levels of TLR5 and TLR7 transcription in hepatic tissue were consistently significantly greater than those in spleen and PBMC of healthy animals after adjustment to either β-actin or HPRT expression. This was in contrast to expression of the same TLRs in humans where transcription levels were significantly higher in spleen and PBMC than in livers (Zarember and Godowski, 2002; Nishimura and Naito, 2005). However, similar to human samples, TLR1 was expressed at significantly greater levels in woodchuck PBMC and spleen than in livers or isolated hepatocytes when adjusted to β-actin or HPRT (see Supplementary Figure S3 and Figure 1, respectively). The differences in expression of TLRs at basal (i.e., healthy state) level between woodchucks and humans could be attributed to species-specific differences, as reported in other species (Rehli, 2002; Heinz et al., 2003; Vaure and Liu, 2014; Perkins and Vogel, 2016). Overall, our study defined TLR 1–10 gene expressional profiles in healthy woodchucks. This assessment established baselines for comparative analyses of data from WHV-infected animals. The relative limitation of our work was its focus on evaluation of the TLR 1–10 genes’ transcription. However, this type of analysis has been typically the first step in recognition of contributions of different TLRs to immunological responses and disease processes, and frequently proven to be a highly valuable source of information that prompted further research (e.g., Applequist et al., 2002; Zarember and Godowski, 2002; Werling et al., 2017). In the case of the woodchuck-WHV model, characterization of expression of multiple TLR proteins and their responses to ligands and antagonists is not yet fully feasible due to the lack of the majority of specific antibodies. This gap will be likely filled up with time and together with the transcriptional profiles delineated in this study provide more comprehensive picture on the TLR involvement in the development, progression and cessation of various forms of liver inflammation coinciding with hepadnaviral infection.

The finding of significant differences in the TLR expression profiles between livers and hepatocytes in some groups of infected woodchucks was rather unexpected. This was somewhat surprising since livers of healthy woodchucks and hepatocytes isolated from them did no show significant differences in the TLRs’ transcription, except TLR7; although non-significant increases in the mean expression of other TLRs in hepatocytes over livers were noted. Thus, when comparing TLRs’ expression in paired livers and hepatocytes from animals with SLAH/SOI, significantly lower mean levels of TLR3, TLR4, and TLR10 were identified in whole hepatic tissue than hepatocytes, while mean TLR7 expression was significantly higher in livers than hepatocytes (see Figure 3). Furthermore, hepatocytes from SLAH/SOI displayed significantly greater mean transcription levels of TLR3, TLR7, and TLR10 than hepatocytes from CH. As indicated before, diagnosis of SLAH is considered when serum WHsAg clears and biochemical markers of liver injury normalize. This is accompanied by elimination or substantial reduction of liver inflammation, as evidenced by histological and immunohistochemical examinations, while WHV replication persists for life at very low levels in both hepatocytes and the immune system (Michalak et al., 1999; Guy et al., 2008). In contrast, a consistent feature of CH is serum WHsAg-positivity, high WHV loads in circulation and hepatocytes, and liver necroinflammation associated with a variable degree of immune cell infiltrations. These infiltrations are mainly composed of T lymphocytes, which express mRNA and protein of most TLRs (Rahman et al., 2009). In addition, B cells, monocytes and NK and NK T cells may occur and they also express TLRs at relatively high levels (Zarember and Godowski, 2002; Tu et al., 2008). In this context, it might not be surprising that expression of certain TLRs in SLAH/SOI was lower in liver tissue than hepatocytes, as inflammatory immune cell infiltration has declined, while hepatocytes in SLAH/SOI transcribed TLRs at greater levels than those in CH in which viral replication and load are high. In this regard, it has been reported that HBV is able to suppress TLR-mediated pro-inflammatory and antiviral innate activities in hepatocytes and non-parenchymal liver cells in mice (Wu et al., 2009a,b). However, it remained unknown whether virus directly inhibits TLR gene expression or acts on TLR signaling or other downstream pathways. The data from the current study tend to suggest a direct effect of virus on TLRs’ transcription, since the event occurred only in hepatocytes during CH, but not SLAH/SOI where WHV occurs at very low levels (see Supplementary Table S1) (Michalak et al., 1999). This may imply that a high-grade of virus replication or its high intracellular load may suppress TLRs. Another impacting factor could be the degree and sites of WHV DNA integration into the hepatocellular genome (Wei et al., 1992; Chauhan et al., 2017), as it was alluded to, but not proved before (Wu et al., 2009a).

In an attempt to gain a better understanding of the TLR transcriptional changes throughout the different stages of WHV infection and hepatitis, serial liver biopsies collected from animals prior to and after virus infection leading to AH followed by CH or SLAH/SOI or after infection with very low doses of virus causing POI were investigated. Detailed analysis of the TLR expression profiles showed that when comparing pre-AH, SLAH/SOI, and POI forms of infection and the healthy state to AH and CH there was significant upregulation of almost all TLRs in the livers from AH and CH stages (see Figure 4). Intrahepatic inflammatory cell infiltrations peak at the time of AH and continue during the course of CH in both humans and woodchucks (Chisari and Ferrari, 1995; Hodgson and Michalak, 2001; Guy et al., 2008; Mani and Kleiner, 2009). In the current study, there was no apparent relation between upregulated TLR expression during AH and caseation of progression to CH, as shown for two animals in Supplementary Figure S5. This raises a possibility that other factors or receptors of the innate immune response may be more important for resolution of hepadnaviral AH. In this regard, our previous studies in the woodchuck model showed that resolution of WHV-induced AH is predetermined by a significantly greater hepatic expression of interferon gamma (IFNγ) and CD3, an increased tumor necrosis alpha (TNFα) transcription, low hepatic WHV load, and a greater degree of liver inflammation than those in acute infection with CH outcome (Hodgson and Michalak, 2001).

In liver biopsies from patients with CH type B, inflammatory infiltrates contain around 75% T cells, 10% B cells and 10% NK cells (Mani and Kleiner, 2009). This strongly suggests that the augmented TLR expression in liver during CH is most likely a consequence of increased TLR transcription in immune cell infiltrates that occurs in the context of downregulated transcription of the majority of TLRs in hepatocytes. Interestingly, TLR1 was detectable in PBMC but not in liver or hepatocytes and there was no significant difference in its expression during the course of WHV infection. However, it is feasible that TLR1 may be more functionally active as an innate immune receptor in the periphery than in the intrahepatic inflammatory infiltrates.

Activation of the innate immune system through manipulating TLR signaling has been a focus in the development of novel treatments against chronic viral infections, including viral hepatitis. In the woodchuck model, the majority of studies were directed toward manipulation of signaling of individual TLRs. However, this took place with a lack of recognition of the complexity of TLRs’ expressional interdependences in the liver and expressional analysis of individual TLR genes in healthy animals and in the course of WHV infection. Our study is the first to delineate the profiles of TLR 1–10 gene transcription in the liver, hepatocytes and PBMC in healthy woodchucks and those with different forms of infection and stages of hepatitis. Similarly, this study is the first to determine TLR1–10 expressional changes chronologically in serial liver biopsies and PBMC obtained throughout the course of WHV infection. The findings indicate that restoration of hepatocyte TLR expression may be essential in resolving CH. Restoring hepatocyte TLR function may promote viral clearance and, in consequence, limit intrahepatic immune cell infiltration and coinciding liver damage. Overall, the findings of this study advance the understanding of the role of TLRs in the course of hepadnaviral infection, particularly during CH. They may contribute to the development of novel antiviral treatments to allow for better control of the virus through a stronger enhancement of intra-hepatocyte innate immune response. Assessment of hepatic TLR expression could also serve as an important biomarker to predict the efficacy of test antiviral approaches and their effectiveness in resolving CH.

## Conclusion

This study provides the first comprehensive determination of TLR 1–10 expression profiles over the course of hepadnaviral infection and in different forms of hepatitis in the woodchuck HBV infection model. Analysis of serial liver biopsy and PBMC samples and autopsy material demonstrated significantly different TLRs’ expression patterns in distinct forms of hepadnaviral infection and stages of hepatitis. It was also uncovered that the TLR expression signatures displayed by liver tissue do not follow those of hepatocytes in some stages of hepatitis, particularly after resolution of acute infection when secondary occult infection endures, and those of peripheral blood mononuclear cells do not mirror accurately those of livers. These findings should be of importance for the dissection of immune process operating at different compartments naturally targeted by hepadnavirus infection and for evaluation of therapies modifying antiviral innate responses in the woodchuck model of hepatitis B and liver cancer.

## Author Contributions

TM, JW, and PM-C conceived and designed the experiments and analyzed the data. JW and AH performed the experiments. TM contributed reagents, materials and analysis tools. JW and TM wrote the paper.

## Conflict of Interest Statement

The authors declare that the research was conducted in the absence of any commercial or financial relationships that could be construed as a potential conflict of interest.

## Footnotes

1 http://www.ncbi.nlm.nih.gov/nuccore

## References

[B1] Aderen A. Ulevitz R. J. (2000). Toll-like receptors in the induction of the innate immune response. *Nature* 406 782–787. 10.1038/35021228 10963608

[B2] Akbal E. Kocak E. Koklu S. Ergul B. Akyurek O. Yilmaz F. M. (2017). Serum toll-like receptor-2, toll-like receptor-4 levels in patients with HBeAg-negative chronic viral hepatitis B. *Viral Immunol.* 30 278–282. 10.1089/vim.2016.0131 28414577 PMC5421507

[B3] Applequist S. E. Wallin R. P. Ljunggren H. G. (2002). Variable expression of Toll-like receptor in murine innate and adaptive immune cell lines. *Int. Immunol.* 14 1065–1074. 10.1093/intimm/dxf069 12202403

[B4] Blasius A. L. Beutier B. (2010). Intracellular Toll-like receptors. *Immunity* 32 305–315. 10.1016/j.immuni.2010.03.012 20346772

[B5] Boni C. Vecchi A. Rossi M. Laccabue D. Giuberti T. Alfieri A. (2018). TLR7 agonist increases responses of hepatitis B virus-specific T cells and natural killer cells in patients with chronic hepatitis B treated with nucleos(t)ide analogues. *Gastroenterology* 154 1764–1777. 10.1053/j.gastro.2018.01.030 29378197

[B6] Chauhan R. Churchill N. D. Mulrooney-Cousins P. M. Michalak T. I. (2017). Initial sites of hepadnavirus integration into host genome in human hepatocytes and in the woodchuck model of hepatitis B-associated hepatocellular carcinoma. *Oncogenesis* 6:e317. 10.1038/oncsis.2017.22 28414318 PMC5520499

[B7] Chen J. Rider D. A. Ruan R. (2006). Identification of valid housekeeping genes and antioxidant enzyme gene expression change in the aging rat liver. *J. Gerontol. Series A Biol. Sci. Med. Sci.* 61 20–27. 16456191 10.1093/gerona/61.1.20

[B8] Chisari F. V. Ferrari C. (1995). Hepatitis B virus immunopathology. *Springer Sem. Immunopathol.* 17 261–281. 10.1007/BF00196169 8571172

[B9] Churchill N. D. Michalak T. I. (2004). Woodchuck hepatitis virus hepatocyte culture models. *Methods Mol. Med.* 96 175–187. 10.1385/1-59259-670-3:175 14762269

[B10] Coffin C. S. Pham T. N. Q. Mulrooney P. M. Churchill N. D. Michalak T. I. (2004). Persistence of isolated antibodies to woodchuck hepatitis virus core antigen is indicative of occult infection. *Hepatology* 40 1053–1061. 10.1002/hep.20419 15382154

[B11] Diao J. Churchill N. D. Michalak T. I. (1998). Complement-mediated cytotoxicity and inhibition of ligand binding to hepatocytes by woodchuck hepatitis virus-induced autoantibodies to asialoglycoprotein receptor. *Hepatology* 27 1623–1631. 10.1002/hep.510270623 9620336

[B12] Gane E. J. Lim Y. S. Gordon S. C. Visvanathan K. Sicard E. Fedorak R. N. (2015). The oral toll-like receptor-7 agonist GS-9620 in patients with chronic hepatitis B virus infection. *J. Hepatol.* 63 320–328. 10.1016/j.jhep.2015.02.037 25733157

[B13] Ganem D. Prince A. M. (2004). Hepatitis B virus infection – natural history and clinical consequences. *New Engl. J. Med.* 350 1118–1129. 10.1056/NEJMra031087 15014185

[B14] Gujar S. A. Jenkins A. K. Guy C. S. Wang J. Michalak T. I. (2008). Aberrant lymphocyte activation precedes delayed virus-specific T-cell response after both primary infection and secondary exposure to hepadnavirus in the woodchuck model of hepatitis B virus infection. *J. Virol.* 82 6992–7008. 10.1128/JVI.00661-08 18480439 PMC2446969

[B15] Gujar S. A. Michalak T. I. (2005). Flow cytometric quantification of T cell proliferation and division kinetics in woodchuck model of hepatitis B. *Immunol. Invest.* 34 215–236. 10.1081/IMM-55834 15921160

[B16] Gujar S. A. Michalak T. I. (2009). Primary occult hepadnavirus infection induces virus-specific T-cell and aberrant cytokine responses in the absence of antiviral antibody reactivity in the Woodchuck model of hepatitis B virus infection. *J. Virol.* 83 3861–3876. 10.1128/JVI.02521-08 19193791 PMC2663258

[B17] Gujar S. A. Mulrooney-Cousins P. M. Michalak T. I. (2013). Repeated exposure to trace amounts of woodchuck hepadnavirus induces molecularly evident infection and virus-specific T cell response in the absence of serological infection markers and hepatitis. *J. Virol.* 87 1035–1048. 10.1128/JVI.01363-12 23135718 PMC3554046

[B18] Guy C. S. Mulrooney-Cousins P. M. Churchill N. D. Michalak T. I. (2008). Intrahepatic expression of genes affiliated with innate and adaptive immune responses immediately after invasion and during acute infection with woodchuck hepadnavirus. *J. Virol.* 82 8579–8591. 10.1128/JVI.01022-08 18596101 PMC2519695

[B19] Guy C. S. Rankin S. L. Michalak T. I. (2011). Hepatocyte cytotoxicity is facilitated by asialoglycoproteon receptor. *Hepatology* 54 1043–1050. 10.1002/hep.24477 21656538

[B20] Guy C. S. Wang J. Michalak T. I. (2010). Hepadnaviral infection augments hepatocyte cytotoxicity mediated by both CD95 ligand and perforin pathways. *Liver Int.* 30 396–405. 10.1111/j.1478-3231.2009.02168.x 19912529

[B21] Heinz S. Haehnel V. Karaghiosoff M. Schwarzfischer L. Muller M. Krause S. W. (2003). Species-specific regulation of Toll-like receptor 3 genes in men and mice. *J. Biol. Chem.* 278 21502–21509. 10.1074/jbc.M301476200 12672806

[B22] Hodgson P. D. Michalak T. I. (2001). Augmented hepatic interferon gamma expression and T-cell influx characterize acute hepatitis progressing to recovery and residual lifelong virus persistence in experimental adult woodchuck hepatitis virus infection. *Hepatology* 34 1049–1059. 10.1053/jhep.2001.29004 11679978

[B23] Hua Z. Hou B. (2013). TLR signaling in B-cell development and activation. *Cell Mol. Immunol.* 10 103–106. 10.1038/cmi.2012.61 23241902 PMC4003046

[B24] Korba B. E. Wells F. V. Baldwin B. Cote P. J. Tennant B. C. Popper H. (1989). Hepatocellular carcinoma in woodchuck hepatitis virus infected woodchucks. Presence of viral DNA in tumor tissue from chronic carriers and animals serologically recovered from acute infections. *Hepatology* 9 461–470. 10.1002/hep.1840090321 2465987

[B25] Levrero M. Zucman-Rossi J. (2016). Mechanisms of HBV-induced hepatocellular carcinoma. *J. Hepatol.* 64 S84–S101. 10.1016/j.jhep.2016.02.021 27084040

[B26] Mani H. Kleiner D. E. (2009). Liver biopsy findings in chronic hepatitis B. *Hepatology* 49 S61–S71. 10.1002/hep.22930 19399798

[B27] McMahon B. J. (2010). Natural history of chronic hepatitis B. *Clin. Liver Dis.* 14 381–396. 10.1016/j.cld.2010.05.007 20638020

[B28] Mencin A. Kluwe J. Schwabe R. F. (2009). Toll-like receptors as targets in chronic liver disease. *Gut* 58 704–720. 10.1136/gut.2008.156307 19359436 PMC2791673

[B29] Meng Z. Zhang X. Pei R. Zhang E. Kemper T. Vollmer J. (2016). Combination therapy including CpG oligodeoxynucleotides and entecavir induces early viral response and enhanced inhibition of viral replication in a woodchuck model of chronic hepadnaviral infection. *Antiviral Res.* 125 14–24. 10.1016/j.antiviral.2015.11.001 26585244

[B30] Menne S. Cote P. J. (2007). The woodchuck as an animal model for pathogenesis and therapy of chronic hepatitis B virus infection. *World J. Gastroenterol.* 13 104–124. 10.3748/wjg.v13.i1.104 17206759 PMC4065868

[B31] Menne S. Tumas D. B. Liu K. H. Thampi L. Aldeghaither D. Baldwin B. H. (2015). Sustained efficacy and seroconversion with the Toll-like receptor 7 agonist GS-9620 in the woodchuck model of chronic hepatitis B. *J. Hepatol.* 62 1237–1245. 10.1016/j.jhep.2014.12.026 25559326 PMC4439359

[B32] Michalak T. I. (1998). The woodchuck animal model of hepatitis B. *Viral Hepatitis Rev.* 4 139–165.

[B33] Michalak T. I. (2000). Occult persistence and lymphotropism of hepadnaviral infection: insights from the woodchuck viral hepatitis model. *Immunol. Rev.* 174 98–111. 10.1034/j.1600-0528.2002.017406.x 10807510

[B34] Michalak T. I. Lin B. (1994). Molecular species of hepadnavirus core and envelope polypeptides in hepatocyte plasma membrane of woodchucks with acute and chronic viral hepatitis. *Hepatology* 20 275–286. 8045487

[B35] Michalak T. I. Mulrooney P. M. Coffin C. S. (2004). Low doses of hepadnavirus induce infection of the lymphatic system that does not engage the liver. *J. Virol.* 78 1730–1738. 10.1128/JVI.78.4.1730-1738.2004 14747538 PMC369489

[B36] Michalak T. I. Pardoe I. U. Coffin C. S. Churchill N. D. Freake D. S. Smith P. (1999). Occult lifelong persistence of infectious hepadnavirus and residual liver inflammation in woodchucks convalescent from acute viral hepatitis. *Hepatology* 29 928–938. 10.1002/hep.510290329 10051500

[B37] Michalak T. I. Pasquinelli C. Guilhot S. Chisari F. V. (1994). Hepatitis B virus persistence after recovery from acute viral hepatitis. *J. Clin. Invest.* 93 230–239. 10.1172/JCI116950 8282792 PMC293757

[B38] Michalak T. I. Snyder R. L. Churchill N. D. (1989). Characterization of the incorporation of woodchuck hepatitis virus surface antigen into hepatocyte plasma membrane in woodchuck hepatitis and in the virus-induced hepatocellular carcinoma. *Hepatology* 10 44–55. 10.1002/hep.1840100111 2535620

[B39] Mulrooney-Cousins P. M. Chauhan R. Churchill N. D. Michalak T. I. (2014). Primary seronegative but molecularly evident hepadnaviral infection engages liver and induces hepatocarcinoma in the woodchuck model of hepatitis B. *PLoS Pathog.* 10:e1004332. 10.1371/journal.ppat.1004332 25165821 PMC4148403

[B40] Mulrooney-Cousins P. M. Michalak T. I. (2015). Asymptomatic hepadnaviral persistence and its consequences in the woodchuck model of occult hepatitis B virus infection. *J. Clin. Transl. Hepatol.* 3 211–219. 10.14218/JCTH.2015.00020 26623268 PMC4663203

[B41] Nassal M. (2008). Hepatitis B viruses: reverse transcription a different way. *Virus Res.* 134 235–249. 10.1016/j.virusres.2007.12.024 18339439

[B42] Nishimura M. Naito S. (2005). Tissue-specific mRNA expression profiles of human toll-like receptors and related genes. *Biol. Pharm. Bull.* 28 886–892. 10.1248/bpb.28.886 15863899

[B43] Niu C. Li L. Daffis S. Lucifora J. Bonnin M. Maadadi S. (2018). Toll-like receptor 7 agonist GS-9620 induces prolonged inhibition of HBV via a type I interferon-dependent mechanism. *J. Hepatol.* 68 922–931. 10.1016/j.jhep.2017.12.007 29247725

[B44] Perkins D. J. Vogel S. N. (2016). Species-specific TLR signaling - insight into human disease. *Nat. Rev. Rheumatol.* 12 198–200. 10.1038/nrrheum.2016.36 27006311 PMC4955569

[B45] Pham T. N. Q. MacParland S. A. Mulrooney P. M. Cooksley H. Naoumov N. V. Michalak T. I. (2004). Hepatitis C virus persistence after spontaneous or treatment-induced resolution of hepatitis C. *J. Virol.* 78 5867–5874. 10.1128/JVI.78.11.5867-5874.2004 15140984 PMC415836

[B46] Plain K. M. Purdie A. C. Begg D. J. De Silva K. Whittington R. J. (2010). Toll-like receptor (TLR)6 and TLR1 differentiation in gene expression studies of Johne’s disease. *Vet. Immunol. Immunopathol.* 137 142–148. 10.1016/j.vetimm.2010.04.002 20434222

[B47] Popper H. Shih J. W. Gerin J. L. Wong D. C. Hoyer B. H. London W. T. (1981). Woodchuck hepatitis and hepatocellular carcinoma: correlation of histologic with virologic observations. *Hepatology* 1 91–98. 10.1002/hep.1840010202 6269981

[B48] Rahman A. H. Taylor D. K. Turka L. A. (2009). The contribution of direct TLR signaling to T cell responses. *Immunol. Res.* 45 25–36. 10.1007/s12026-009-8113-x 19597998 PMC4486050

[B49] Raimondo G. Allain J.-P. Brunetto M. R. Buendia M.-A. Chen D.-S. Colombo M. (2008). Statements from Taormina expert meeting on occult hepatitis B virus infection. *J. Hepatol.* 49 652–657. 10.1016/j.jhep.2008.07.014 18715666

[B50] Rehli M. (2002). Of mice and men: species variations of Toll-like receptor expression. *Trends Immunol.* 23 375–378. 10.1016/S1471-4906(02)02259-7 12133792

[B51] Rock F. L. Hardiman G. Timans J. C. Kastelein R. A. Bazan J. F. (1998). A family of human receptors structurally related to Drosophila Toll. *Proc. Natl. Acad. Sci. U.S.A.* 95 588–593. 10.1073/pnas.95.2.588 9435236 PMC18464

[B52] Roggendorf M. Kosinska A. D. Liu J. Lu M. (2015). The woodchuck, a nonprimate model for immunopathogenesis and therapeutic immunomodulation in chronic hepatitis B virus infection. *Cold Spring Harb. Perspect. Med.* 5:021451. 10.1101/cshperspect.a021451 26511761 PMC4665037

[B53] Ryerson A. B. Eheman C. R. Altekruse S. F. Ward J. W. Jemal A. Sherman R. L. (2016). Annual report to the nation on the status of cancer, 1975-2012, featuring the increasing incidence of liver cancer. *Cancer* 122 1312–1337. 10.1002/cncr.29936 26959385 PMC4840031

[B54] Seeger C. Mason W. S. (2015). Molecular biology of hepatitis B virus infection. *Virology* 47 672–686. 10.1016/j.virol.2015.02.031 25759099 PMC4424072

[B55] Tennant B. C. Toshkov I. A. Peek S. F. Jacob J. R. Menne S. Hornbuckle W. E. (2004). Hepatocellular carcinoma in the woodchuck model of hepatitis B virus infection. *Gastroenterology* 127 S283–S293. 10.1053/j.gastro.2004.09.043 15508096

[B56] Tsaur I. Renninger M. Hennenlotter J. Oppermann E. Munz M. Kuehs U. (2013). Reliable housekeeping gene combination for quantitative PCR of lymph nodes in patients with prostate cancer. *Anticancer. Res.* 33 5243–5248. 24324056

[B57] Tu Z. Bozorgzadeh A. Pierce R. H. Kurtis J. Crispe I. N. Orloff M. S. (2008). TLR-dependent cross talk between human Kupffer cells and NK cells. *J. Exp. Med.* 205 233–244. 10.1084/jem.20072195 18195076 PMC2234385

[B58] Vaure C. Liu Y. (2014). A comparative review of toll-like receptor 4 expression and functionality in different animal species. *Front. Immunol.* 5:316. 10.3389/fimmu.2014.00316 25071777 PMC4090903

[B59] Wei Y. Ponzetto A. Tiollais P. Buendia M.-A. (1992). Multiple rearrangements and activated expression of c-myc induced by woodchuck hepatitis virus integration in a primary liver tumour. *Res. Virol.* 143 89–96. 10.1016/S0923-2516(06)80086-5 1317604

[B60] Werling D. Hope J. C. Siddiqui N. Widdison S. Russell C. Sopp P. (2017). Subset-specific expression of Toll-like receptors by bovine afferent lymph dendritic cells. *Front. Vet. Sci.* 4:44. 10.3389/fvets.2017.00044 28421187 PMC5376590

[B61] World Health Organization (2018). *Hepatitis B Fact Sheet; Updated July 2018.* Geneva: World Health Organization.

[B62] Wu J. Meng Z. Jiang M. Pei R. Trippler M. Broering R. (2009a). Hepatitis B virus suppresses toll-like receptor-mediated innate immune responses in murine parenchymal and nonparenchymal liver cells. *Hepatology* 49 1132–1140. 10.1002/hep.22751 19140219

[B63] Wu J. Meng Z. Jiang M. Zhang E. Trippler M. Broering R. (2009b). Toll-like receptor-induced innate immune responses in non-parenchymal liver cells are cell type-specific. *Immunology* 129 363–374. 10.1111/j.1365-2567.2009.03179.x 19922426 PMC2826681

[B64] Yang J. D. Roberts L. R. (2010). Hepatocellular carcinoma: a global view. *Nat. Rev. Gastroenterol. Hepatol.* 7 448–458. 10.1038/nrgastro.2010.100 20628345 PMC3926946

[B65] Zarember K. A. Godowski P. J. (2002). Tissue expression of human Toll-like receptors and differential regulation of Toll-like receptor mRNAs in leukocytes in response to microbes, their products, and cytokines. *J. Immunol.* 168 554–561. 10.4049/jimmunol.168.2.554 11777946

[B66] Zerbini A. Pilli M. Boni C. Fisicaro P. Penna A. Di Vincenzo P (2008). The characteristics of the cell-mediated immune response identify different profiles of occult hepatitis B virus infection. *Gastroenterology* 134 1470–1481. 10.1053/j.gastro.2008.02.017 18355815

[B67] Zhang X. Ma Z. Liu H. Liu J. Meng Z. Broering R. (2012). Role of Toll-like receptor 2 in the immune response against hepadnaviral infection. *J. Hepatol.* 57 522–528. 10.1016/j.jhep.2012.05.004 22617154

[B68] Zhang X. Meng Z. Qiu S. Xu Y. Yang D. Schlaak J. F. (2009). Lipopolysaccharide-induced innate immune responses in primary hepatocytes downregulates woodchuck hepatitis virus replication via interferon-independent pathways. *Cell. Microbiol.* 11 1624–1637. 10.1111/j.1462-5822.2009.01353.x 19573162

