# Supplementary material for: Differential Expression of Woodchuck Toll-Like Receptors 1–10 in Distinct Forms of Infection and Stages of Hepatitis in Experimental Hepatitis B Virus Infection
Source: Front Microbiol. 2018 Dec 7;9:3007. doi: 10.3389/fmicb.2018.03007 (PMC6292964; doi:10.3389/fmicb.2018.03007)
Supplement: Supplementary file 1 [file Presentation_1.pdf]

Supplementary Material Table 1. The mean WHV levels in plasma, liver and PBMC of woodchucks with different forms of WHV infection and stages of WHV-induced hepatitis examined in this study*

Infection form or hepatitis stage	Animal number**	Gender M/F	Plasma (vge/mL ± SEM)			Liver (vge/µg DNA ± SEM)			PBMC (vge/µg DNA ± SEM)		
Healthy	21	12/9	0.00E+00	±	0.00E+00	0.00E+00	±	0.00E+00	0.00E+00	±	0.00E+00
PreAH	7	1/6	2.17E+04	±	1.31E+04	3.67E+03	±	3.18E+03	3.86E+02	±	1.61E+02
AH	6	3/3	1.36E+08	±	7.15E+07	1.27E+06	±	1.25E+06	3.09E+03	±	1.12E+03
SLAH/SOI	8	2/6	2.33E+03	±	9.58E+02	2.01E+03	±	8.30E+02	8.50E+01	±	1.50E+01
CH***	10	7/3	1.86E+10	±	1.33E+10	2.78E+07	±	9.32E+06	2.62E+03	±	1.85E+03
POI	6	3/3	5.79E+01	±	1.23E+01	9.29E+00	±	7.15E+00	3.00E+01	±	1.36E+01

M, Male; F, female; PreAH, pre-acute hepatitis; AH, acute hepatitis; SLAH/SOI, self-limited acute hepatitis followed by secondary occult infection; CH, chronic hepatitis; POI, primary occult infection.

* Mean WHV DNA load values for plasma and PBMC determined in samples collected at the time of acquisition of liver tissue usually obtained at 6-12 month intervals.

**Cumulative number of animals in which samples WHV load was determined.

***Four (3M/1F) animals developed HCC. Non-tumorous liver vtissue was used for WHV DNA quantification.

Supplementary Material Table 2. Numbers of samples from heathy woodchucks and animals with two opposing forms of experimental WHV hepatitis

Sample	Animal group/Sample number			Total animal/ Sample number
	Healthy (3M/1F)	SLAH/SOI (1M/6F)	CH* (3M/2F)	
Liver	4	7	5	16
Spleen	4	7	5	16
Bone marrow	4	7	5	16
PBMC	4	7	5	16

M, male; F, Female; SLAH/SOI, self-limited acute hepatitis followed by secondary occult WHV infection; CH, serum WHsAg-positive chronic hepatitis

*Two male animals developed HCC. TLRs' expression was quantified in non-tumorous liver tissue.

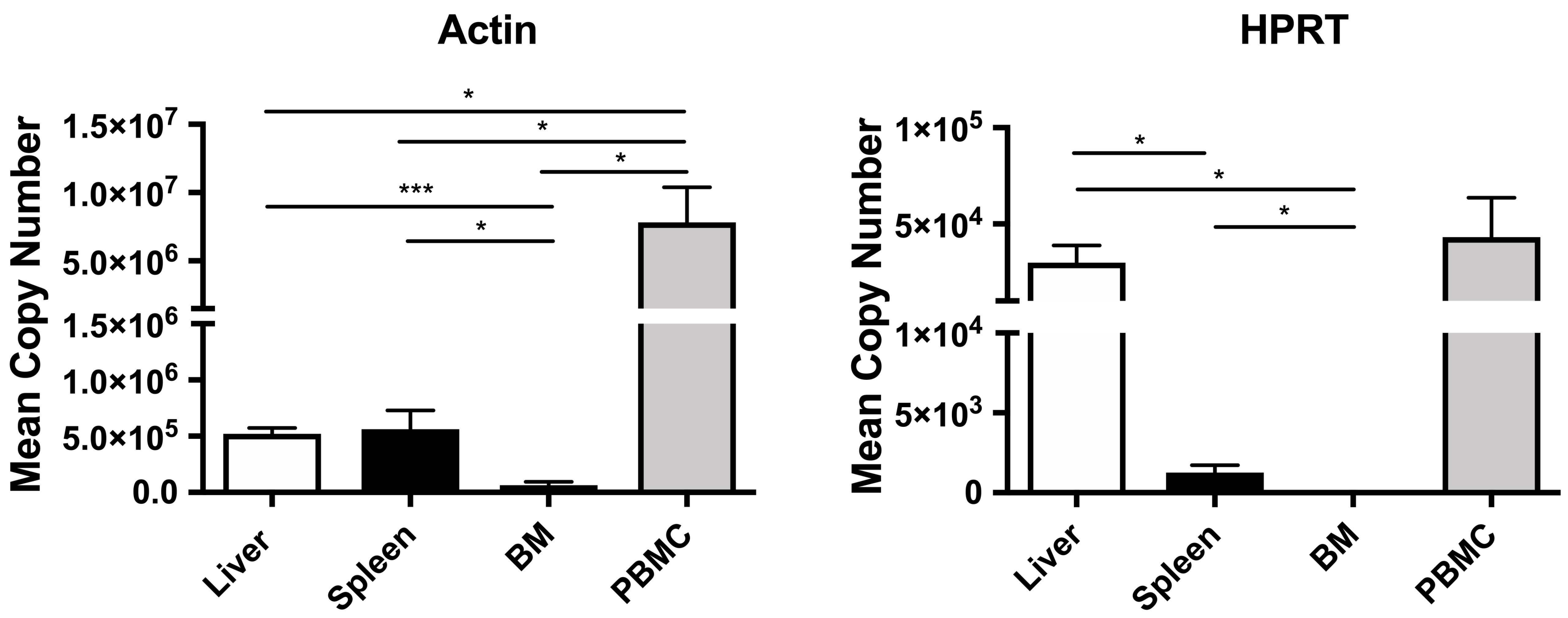

Supplementary Material Figure 1. Comparison of the expression of β-actin **(A)** and HPRT **(B)** in liver, spleen, bone marrow and PBMC samples obtained at autopsy of 4 healthy, adult woodchucks. The transcription levels were evaluated by RT-qPCR with respective woodchuck-specific primer pairs. The data shown are mean expression values ± SEM. Differences between data bars are marked with * are significant at $P < 0.05$.

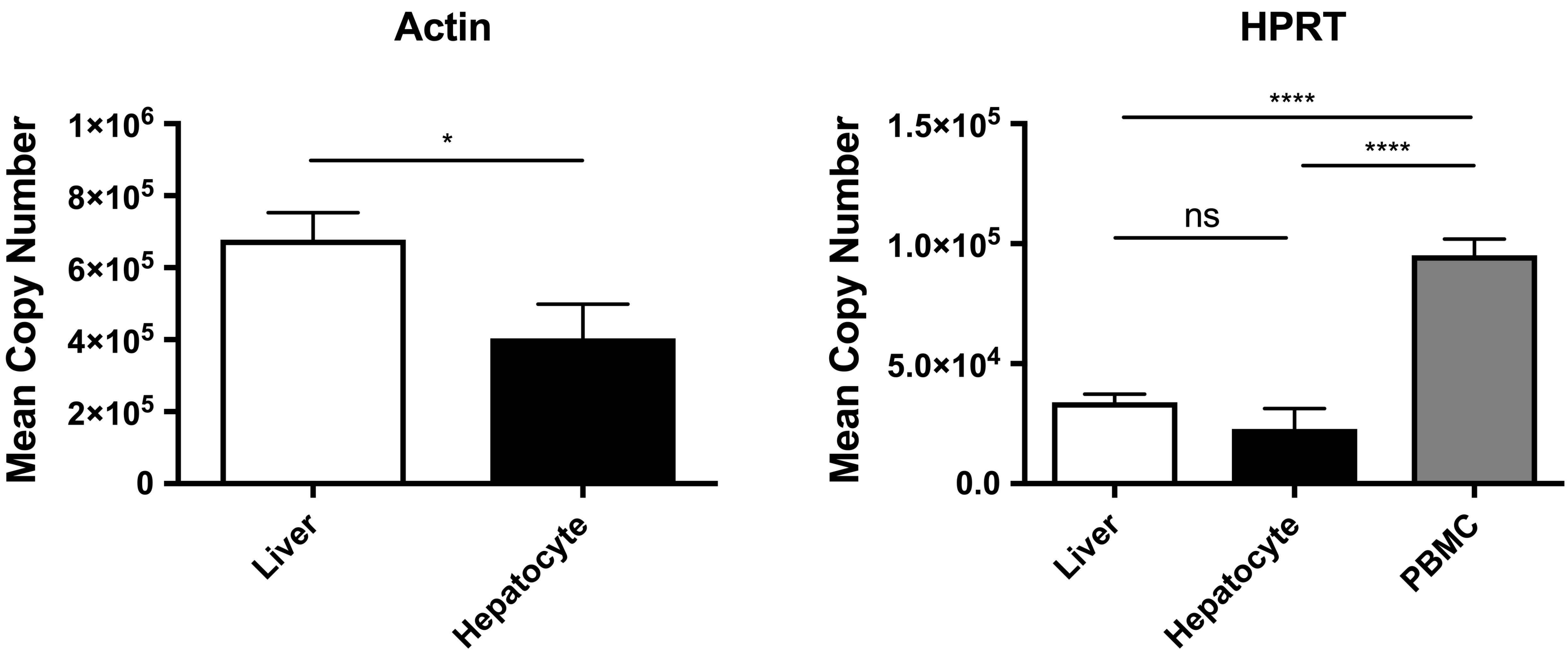

Supplementary Material Figure 2. HPRT is a more reliable housekeeping gene than β-actin when comparing gene expression in woodchuck livers and hepatocytes isolated from these livers. **(A)** Liver samples and hepatocytes obtained from 26 animals prior to and during different forms of WHV infection and WHV-induced hepatitis. β-actin housekeeping gene was expressed at significantly higher mean level in livers than hepatocytes isolated from these livers. **(B)** Liver (n = 87), hepatocyte (n = 26), and PBMC (n = 51) samples were isolated from woodchucks prior to and during WHV infection. HPRT was expressed at similar mean levels in livers and derived hepatocytes (marked as not significant, ns), while its transcription was significantly higher in PBMC. Results are shown as mean values ± SEM. Differences between data bars marked with * are significant at $P < 0.05$, and **** at $P \leq 0.0001$ by two-tailed Student's *t*-test.

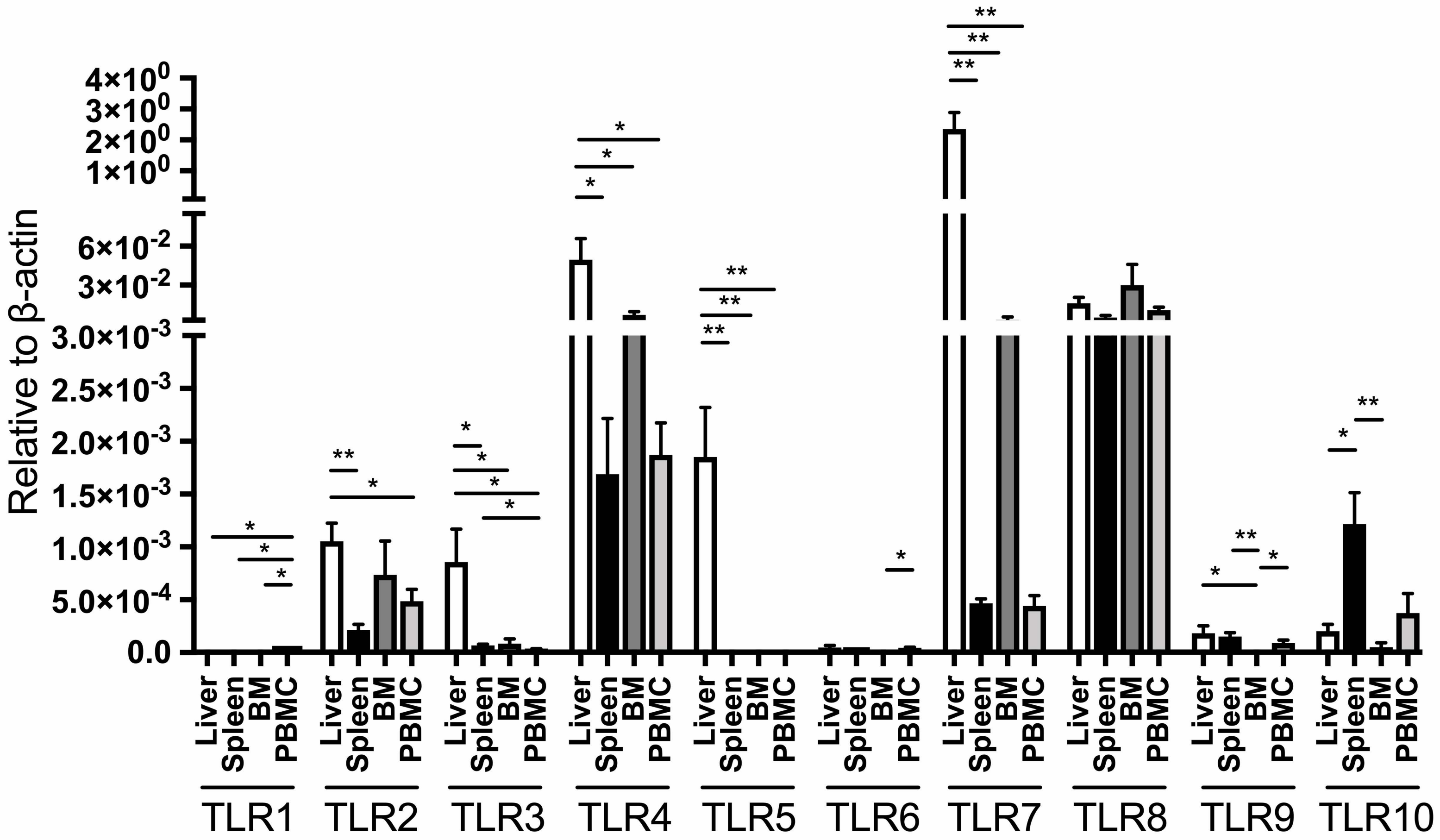

Supplementary Material Figure 3. Distribution of TLR 1-10 expression in liver, spleen, bone marrow and PBMC obtained from 4 healthy adult woodchucks when normalized to expression of woodchuck β-actin instead of HPRT shown in Figure 1. The results are presented as relative mean values ± SEM. Statistically significant differences between data bars are marked with *, ** or *** as explained in Materials and Methods. TLR1 was not detectable in liver, spleen and bone marrow but was at low copy numbers in PBMC.

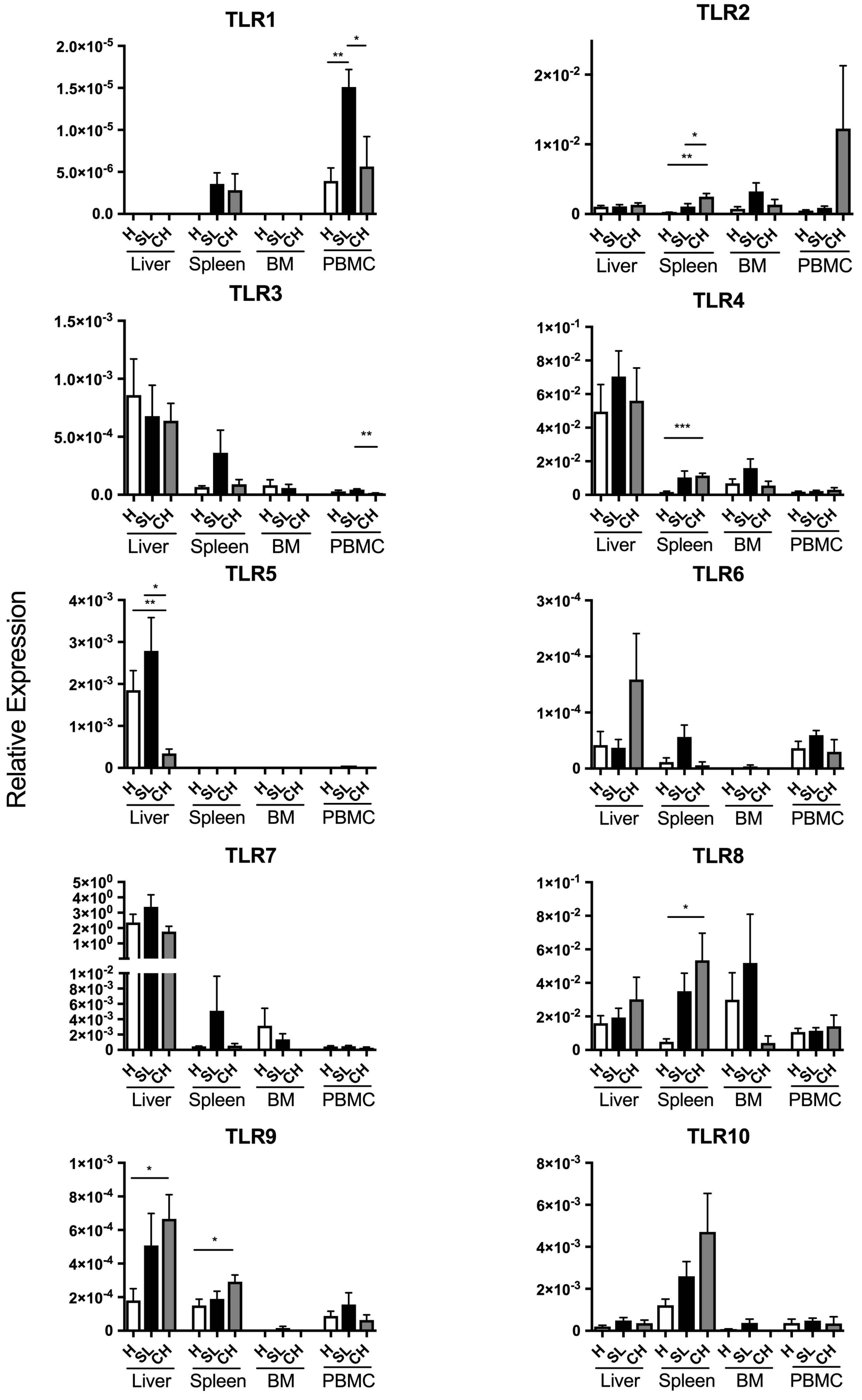

Supplimentary Material Figure 4. Comparison of the expression of TLRs 1-10 in liver, spleen, bone marrow (BM) and PBMC samples obtained at autopsy of woodchucks which spontaneously resolved acute hepatitis (SL; n = 7) or during progression of chronic hepatitis (CH; n = 5) and from healthy animals (H; n = 4). The expression levels were normalized to β-actin and are presented as relative mean values ± SEM. Significant differences between data bars are marked as outlined in Materials and Methods.

A

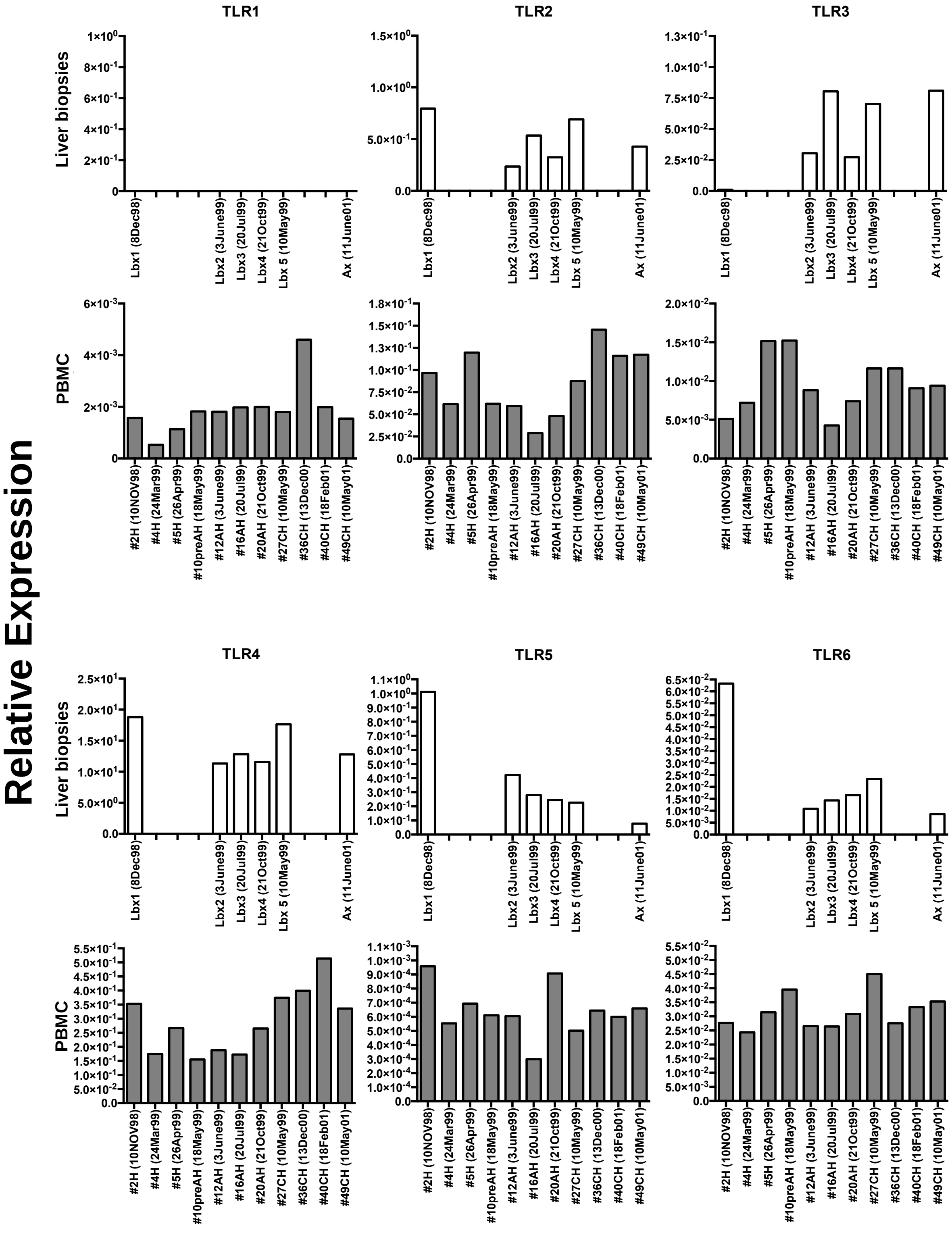

Supplementary Figure 5A

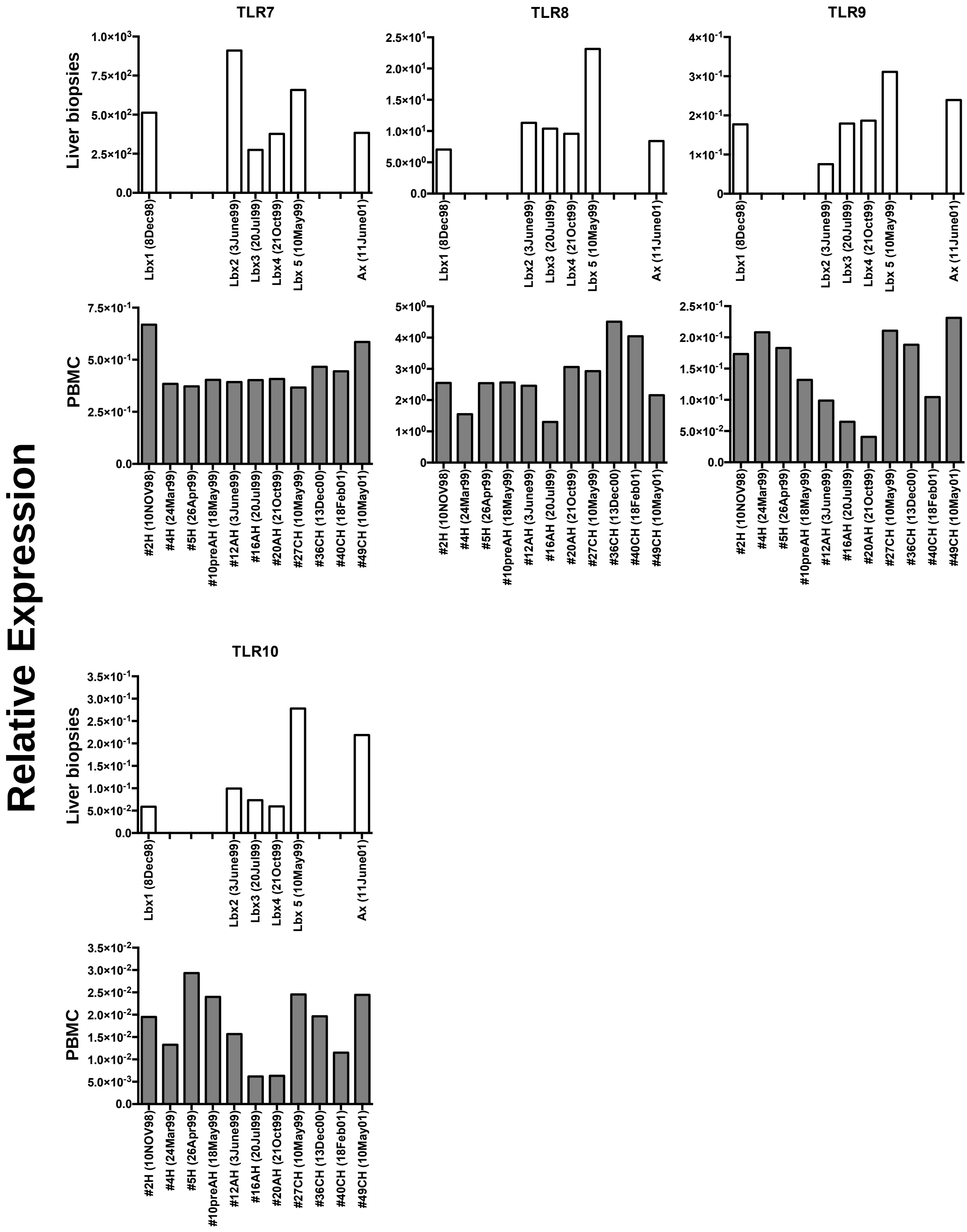

Supplementary Figure 5A

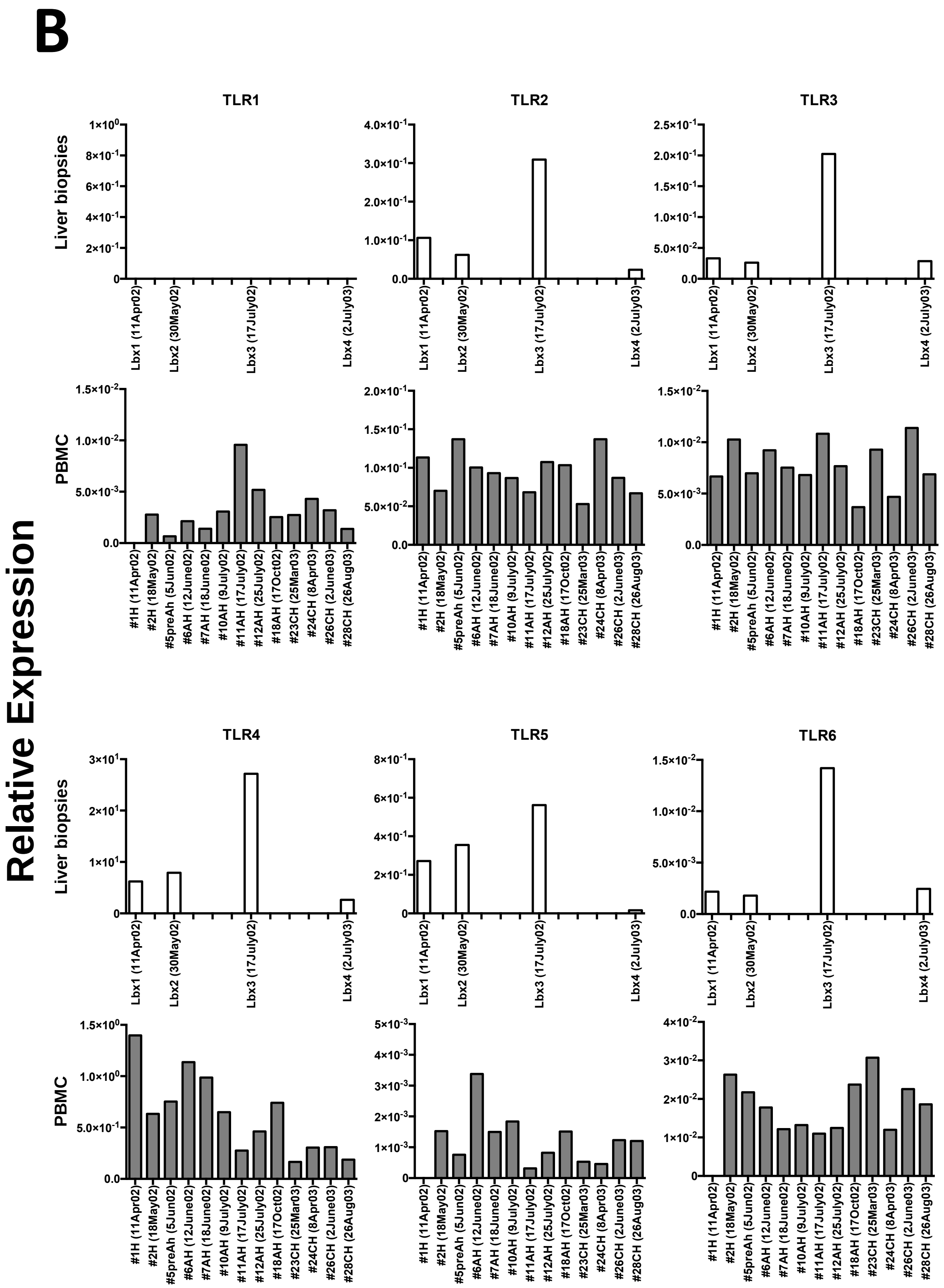

Supplementary Figure 5B

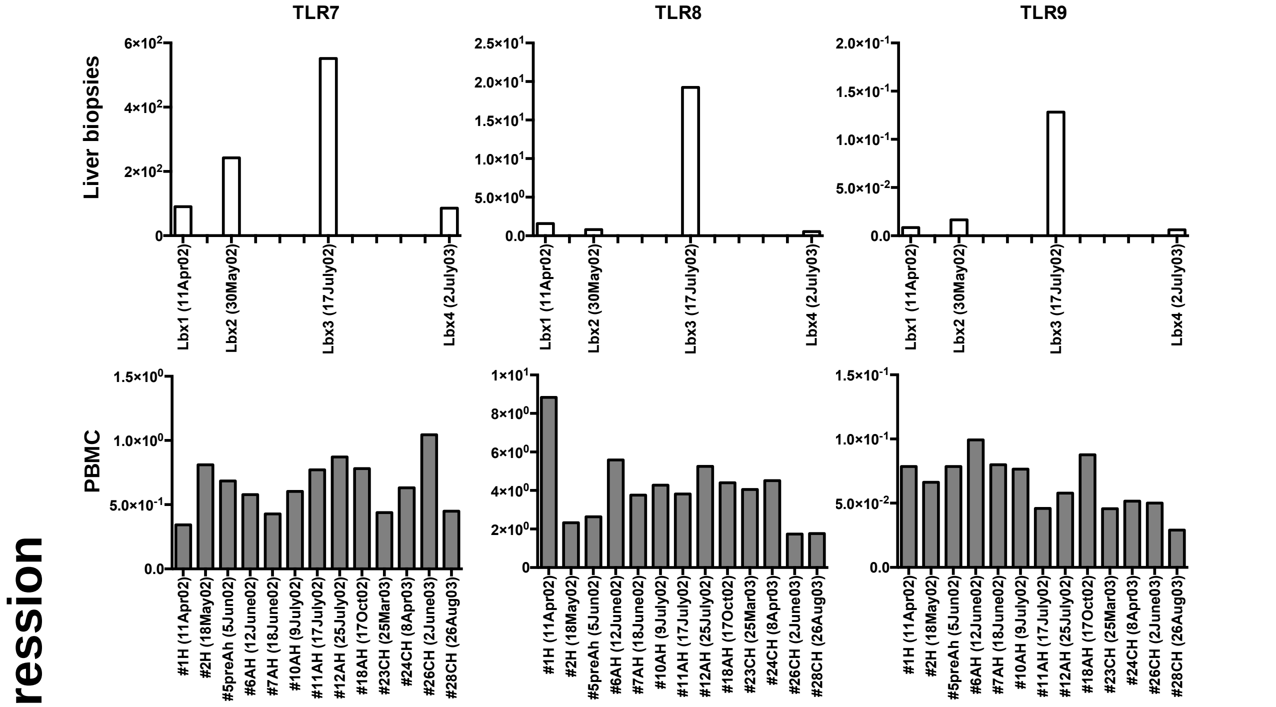

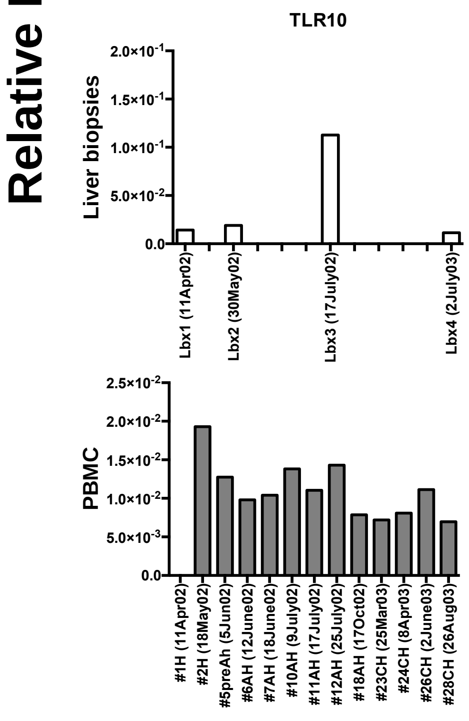

Supplementary Figure 5. Expression levels of TLRs1-10 in sequential liver biopsy and PBMC samples obtained from two woodchucks before inoculation with WHV and during the course of WHV infection that progressed from acute hepatitis (AH) to chronic hepatitis (CH). Liver biopsy tissue samples (top; white bars) and PBMC (bottom; grey bars) isolated from woodchuck #1 **(A)** and woodchuck #2 **(B)**. TLR mRNA was quantified by RT-qPCR. Total RNA equivalent of 50 ng from each sample was quantified in triplicate and the bars represents means of these three determinations. TLR1 expression was evaluated but not detected in the liver. Gene transcription levels were normalized against expression of woodchuck HPRT.

Supplementary Figure 5B
